# Mutations Upstream of the *TBX5* and *PITX1* Transcription Factor Genes Are Associated with Feathered Legs in the Domestic Chicken

**DOI:** 10.1093/molbev/msaa093

**Published:** 2020-04-28

**Authors:** Jingyi Li, MiOk Lee, Brian W Davis, Sangeet Lamichhaney, Ben J Dorshorst, Paul B Siegel, Leif Andersson*

**Affiliations:** m1 Department of Veterinary Integrative Biosciences, College of Veterinary Medicine and Biomedical Sciences, Texas A&M University, College Station, TX; m2 Department of Animal and Poultry Sciences, Virginia Polytechnic Institute and State University, Blacksburg, VA; m3 Science for Life Laboratory, Department of Medical Biochemistry and Microbiology, Uppsala University, Uppsala, Sweden; m4 Department of Animal Breeding and Genetics, Swedish University of Agricultural Sciences, Uppsala, Sweden

**Keywords:** chicken, feathered-leg, linkage mapping, IBD mapping, *TBX5*, *PITX1*

## Abstract

Feathered leg is a trait in domestic chickens that has undergone intense selection by fancy breeders. Previous studies have shown that two major loci controlling feathered leg are located on chromosomes 13 and 15. Here, we present genetic evidence for the identification of candidate causal mutations at these loci. This was accomplished by combining classical linkage mapping using an experimental cross segregating for feathered leg and high-resolution identical-by-descent mapping using whole-genome sequence data from 167 samples of chicken with or without feathered legs. The first predicted causal mutation is a single-base change located 25 kb upstream of the gene for the forelimb-specific transcription factor TBX5 on chromosome 15. The second is a 17.7-kb deletion located ∼200 kb upstream of the gene for the hindlimb-specific transcription factor PITX1 on chromosome 13. These mutations are predicted to activate *TBX5* and repress *PITX1* expression, respectively. The study reveals a remarkable convergence in the evolution of the feathered-leg phenotype in domestic chickens and domestic pigeons, as this phenotype is caused by noncoding mutations upstream of the same two genes. Furthermore, the *PITX1* causal variants are large overlapping deletions, 17.7 kb in chicken and 44 kb in pigeons. The results of the present study are consistent with the previously proposed model for pigeon that feathered leg is caused by reduced *PITX1* expression and ectopic expression of *TBX5* in hindlimb buds resulting in a shift of limb identity from hindlimb to more forelimb-like identity.

## Introduction

The ancestors of chickens had tarsometatarsus and digits that were covered with scales. The transformation of part of those scales into feathers due to genetic changes results in so-called feathered leg, feathered feet, shank feathering, or ptilopody (Bartels 2003). Clean leg (lacking feathers) may in fact be the derived state in birds because many basal birds have feathered legs and the “4-winged” dinosaurs had feathered hindlimbs (Xu and Zhang 2005; Hu et al. 2009; Hone et al. 2010). Feathered leg is an attractive trait in fancy chicken breeding, with leg feathering in such breeds ranging from sparse like in Langshan chickens to extensive like in the Cochin breed (Somes 1992). Poultry geneticists have studied the inheritance of feathered legs since the beginning of the last century (Punnett and Bailey 1918; Dunn and Jull 1927; Warren 1933). Somes (1992), based on the results of test mating, provided genetic evidence for the presence of two independent dominant mutations, denoted *Pti-1* and *Pti-2*, causing feathered legs in domestic chicken. Breeds with more sparsely feathered legs were supposed to be homozygous mutant at the *Pti-1* locus but wild-type (*pti-2/pti-2*) at the second locus. Breeds with more heavily feathered legs like Sultan and Cochin were thought to carry the dominant mutations at both *Pti-1* and *Pti-2* (Somes 1992). A recessive allele (*pti-3*) at a third locus has been suggested to cause leg feathering in the Pavloff chicken (Somes 1990).

The identification of the genetic basis for the transformation of scales to feathers on the legs of chicken provides an opportunity to gain insights on mechanisms controlling development of skin appendages in birds. Previous linkage mapping has shown that feathered legs in the Silkie breed is controlled by a locus on chromosome 13 (Dorshorst et al. 2010), which harbors the *paired-like homeodomain 1* (*PITX1*) gene. Sun et al. (2015) identified two chromosomal regions associated with the feathered-leg phenotype in Beijing You chickens, one consistent with the *PITX1* region on chromosome 13, and the other in a region of chromosome 15 containing five genes including *T-box 5* (*TBX5*).

The domestic pigeon is another avian species that shows similar variation in feathered-leg phenotypes as is present in chickens. Domyan et al. (2016) identified two loci associated with feathered legs in pigeons, *PITX1* and *TBX5*, and reported decreased expression of the hindlimb-specific transcription factor *PITX1* and ectopic expression of the forelimb-specific transcription factor *TBX5* in the hindlimb buds of feathered-legged pigeons. Thus, this suggested a parallel evolution of the feathered-leg phenotype in domestic chickens and pigeons because *PITX1* and *TBX5* are located in the two genomic regions on chromosomes 13 and 15, respectively, associated with feathered legs in chicken. In fact, Domyan et al. (2016) also studied the expression of *TBX5* and *PITX1* in the hindlimb buds of feathered-leg Cochin and Silkie chickens, and found a similar ectopic expression of *TBX5* as in the pigeon but no difference in *PITX1* expression. Thus, although the causal mutation affecting *TBX5* expression in feathered-leg chickens needs to be identified, the association between *PITX1* and feathered leg needs to be confirmed.

Here, a three-generation mapping population was established by crossing Langshan (feathered leg) and Houdan (clean leg) chickens, followed by SNP analyses of pooled samples, linkage mapping, whole-genome sequencing (WGS), and diagnostic testing of a comprehensive collection of chicken populations with or without feathered legs. The aim was to identify the casual mutations underlying the feathered-leg phenotype in chickens.

## Results

### SNP Analysis and Linkage Mapping

**Fig. 1.**
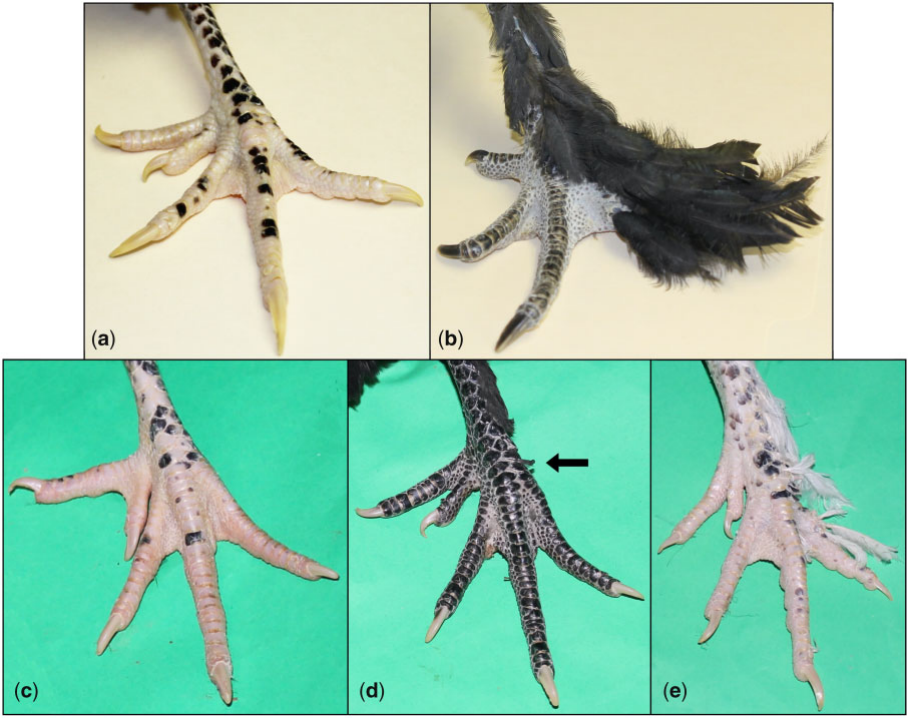
Feathered-leg phenotypes in the mapping population of Houdan (clean leg)×Langshan (feathered leg) cross. (*a*) Purebred Houdan. (*b*) Purebred Langshan. Backcross individuals with clean leg (*c*), intermediate feathered leg (*d*), and feathered leg (*e*). The feather stubs commonly observed in the intermediate phenotype are indicated by an arrow. Photo: Jingyi Li.

A linkage mapping population consisting of wild-type Houdan and feathered-leg Langshan F_0_ birds, 25 F_1_ females, and 222 backcross chickens was established. The latter was generated by crossing F_1_ females with purebred Houdan males. All F_1_ individuals were feathered leg. The segregation of feathered-leg phenotypes in the backcross population was as follows: 82 clean leg (37%), 44 intermediate (20%), and 96 feathered leg (43%) (fig. 1). Backcross individuals expressing feathered leg (fig. 1*e*) have lighter leg feathering than purebred Langshan (fig. 1*b*), suggesting incomplete dominance for the feathered allele in Langshan. Two backcross DNA pools were constructed, Pool_feathered and Pool_clean; the backcross individuals with an intermediate phenotype were not included in these pools. Two parental line DNA pools were also constructed (Pool_Langshan and Pool_Houdan). The two backcross pools were analyzed using a 60k SNP Illumina array, and all four pools were subjected to WGS in order to directly identify candidate causal sequence variants associated with leg feathering.

**Fig. 2.**
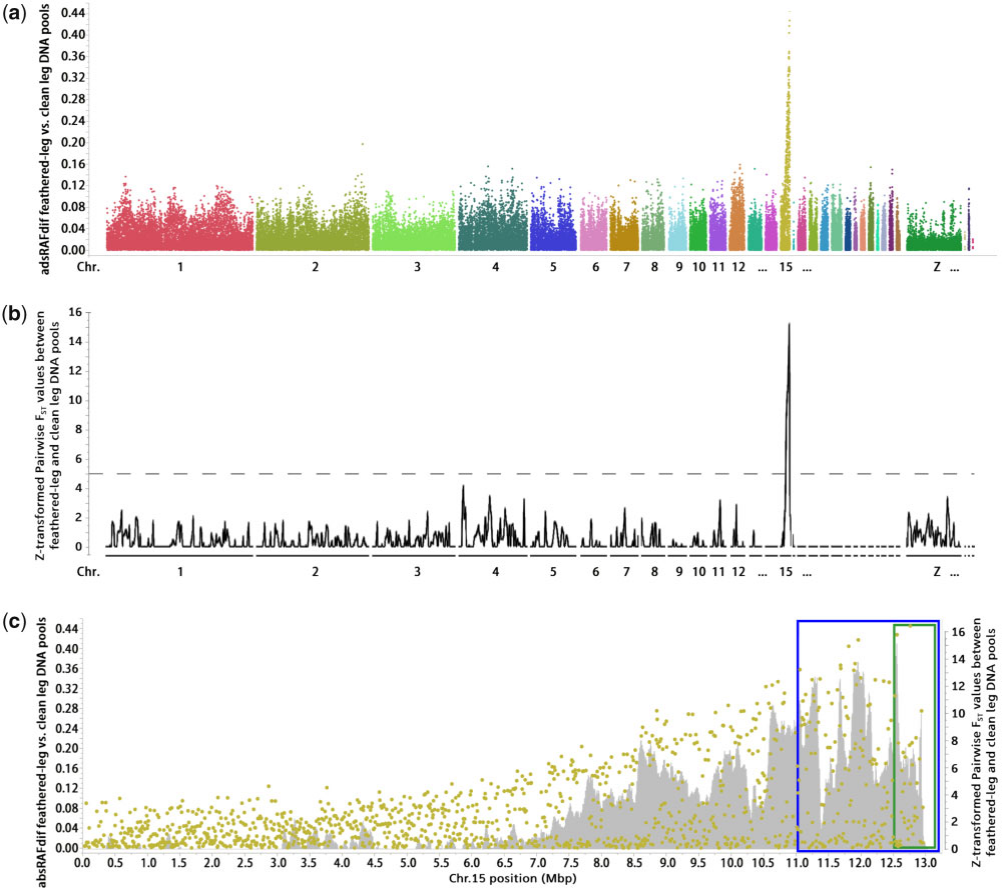
Genetic mapping of the feathered-leg locus on chromosome 15. All the data presented here are based on contrasts between the pools of backcross individuals with feathered leg and clean leg. Genomic positions refer to the GalGal6 assembly. (*a*) Genome-wide absRAFdif values of all 60k SNPs, plotted against their genomic location. (*b*) Genome-wide ZF_ST_ values based on whole-genome sequencing, plotted against their genomic location. (*c*) absRAFdif values for SNPs on chromosome 15 (green dots). All SNPs absRAFdif values >0.35 were located within the 2.05-Mb region (blue box). The green box indicates the 0.56-Mb region defined based on the second round of linkage mapping. Gray area indicates ZF_ST_ values based on whole-genome sequencing.

The maximum possible difference in relative allele frequencies (RAF) between the Pool_feathered and Pool_clean groups is 0.5, which occurs when the parental lines are fixed for different alleles. The analysis of SNP data showed that a region on chromosome 15 from 11.01 to 13.06 Mb (end of the chromosome) contained all SNPs with absolute RAF differences (absRAFdif) values >0.35 (fig. 2*a*). In addition, the analysis of WGS data from the two pools revealed single peaks of high ZF_ST_ values in the same region on chromosome 15 (fig. 2*b*). By genotyping five selected SNPs in all feathered and clean-leg backcross individuals, a first round of linkage mapping revealed that a major locus controlling the feathered-leg phenotype is located in a 1.04-Mb region defined by rs14096198 (11.94 Mb) and the distal end of chromosome 15 (GalGal6 coordinates), completely linked to two SNPs with a log_10_ odds ratio score of 53.6 (supplementary table S1, Supplementary Material online). Only two individuals were recombinant between rs14096198 and the leg feathering locus. These two recombinant individuals were used for a second round of linkage mapping using six more SNPs identified by WGS. These SNPs were fixed for different alleles in the parental lines. As a result, the candidate region was narrowed down to a 0.56-Mb region defined by a SNP at 12,505,096 bp (supplementary table S2, Supplementary Material online and fig. 2*c*) and the distal end of the chromosome. The peak of ZF_ST_ values is also within this 0.56-Mb region (fig. 2*c*).

### Genetic Evidence for a Causal Mutation Upstream of *TBX5* in Most Feathered-Leg Populations

**Fig. 3.**
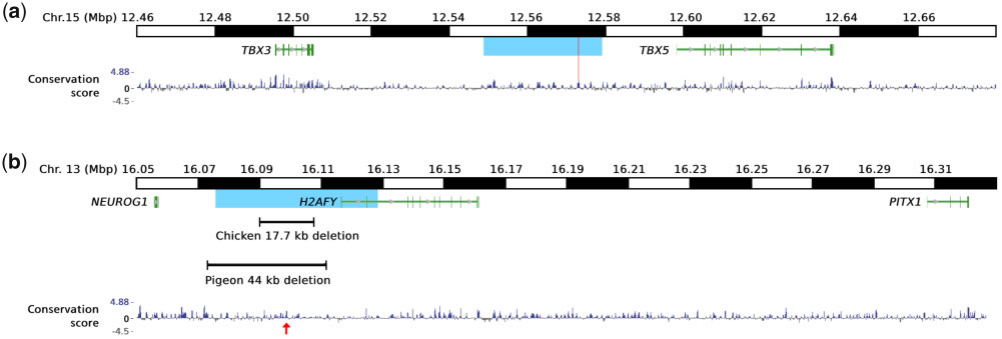
Localization of the major loci on chromosomes 13 and 15 underlying feathered-leg phenotypes in chicken in relation to evolutionarily conserved elements (77 vertebrates basewise PhyloP conservation score, https://hgdownload.soe.ucsc.edu/goldenPath/galGal6/phastCons77way/). Genomic positions refer to the GalGal6 assembly. (*a*) The region on chromosome 15 harboring the *TBX5* gene. The blue area indicates the 30-kb IBD region shared by feathered-leg populations. The red line indicates the predicted causal SNP g.12573054 T>C and its location within a conserved element. (*b*) The region on chromosome 13 harboring the *PITX1* gene. The blue area indicates the 53-kb IBD region shared by some feathered-leg populations. The location of the 17.7-kb deletion as well as the region predicted to correspond to the 44-kb deletion of pigeons with feathered legs (Domyan et al. 2016) and their overlap with conserved elements are indicated. The conserved element showing homology to human hs1473 is indicated by a red arrow.

We analyzed WGS data from eight samples representing seven breeds with feathered legs and 159 samples of chicken lacking feathered leg (supplementary table S3, Supplementary Material online). Analysis of the 0.56-Mb region on chromosome 15 harboring a feathered-leg locus revealed a 30-kb identical-by-descent (IBD) region (chr15:12,548,968–12,579,000 bp, GalGal6) shared by all feathered-leg samples (fig. 3*a*). The results suggest that these eight samples share the same causal mutation(s) associated with the feathered-leg phenotype. Only one sequence change, a SNP (g.12,573,054 T>C), within this IBD region is absent in all 159 clean-leg samples, which provides genetic evidence that this SNP is the causal mutation for the feathered leg locus on chromosome 15. A diagnostic test for this SNP showed, as expected, that all Langshan F_0_ parental birds were homozygous for this mutation, all Houdan F_0_ birds were homozygous wild-type, all F_1_ birds were heterozygous, all backcross individuals with the feathered-leg phenotype were also heterozygous, whereas all clean-leg backcross individuals were homozygous wild-type. There were 44 backcross individuals with an intermediate feathered-leg phenotype, 29 of these had the wild-type T/T genotype, whereas 15 had the mutant C/T genotype at this SNP. The results show that the C/T genotype has a variable phenotype expression in this pedigree and that other genetic factors may cause the intermediate phenotype in the absence of the causal sequence change at the distal end of chromosome 15.

The mutation is located 25 kb upstream of *TBX5* (fig. 3*a*), and within a 297-bp conserved element (CE) based on the 77 vertebrates basewise PhyloP conservation score (https://hgdownload.soe.ucsc.edu/goldenPath/galGal6/phastCons77way/). This 297-bp region in chicken shares 58% sequence identity to the corresponding human sequence (supplementary fig. S1*a*, Supplementary Material online), suggesting its functional importance, possibly as a regulatory element controlling gene expression. TRANSFAC (Kel et al. 2003) analysis predicted that the *TBX5* SNP disrupts the binding sites of the CDX1 (supplementary fig. S1*a*, Supplementary Material online) and MEF2C transcription factors, whereas it is predicted to create new binding sites for GATA3, GATA4, and ZNF233. Possible relationships between each of these transcription factors and *TBX5* expression were explored using existing literature which indicated that only CDX1 is known to regulate *TBX5* expression, and it acts as a repressor of *TBX5* in zebrafish (Lengerke et al. 2011). Interestingly, it has previously been reported that *TBX5* shows ectopic expression in hindlimb buds of feathered-leg chickens (Domyan et al. 2016), consistent with the disruption of the binding site for a *TBX5* repressor.

### Identification of a 17.7-kb Deletion Upstream of *PITX1* in Some Feathered-Leg Populations

Previous studies have provided conclusive evidence for the existence of at least two independent loci controlling the feathered-leg phenotype on chromosomes 13 and 15 (Dorshorst et al. 2010; Sun et al. 2015). The previously defined region on chromosome 15 overlaps perfectly with the results of the present study. However, the second feathered-leg locus on chromosome 13 is apparently not segregating in our intercross (fig. 2*a*). Based on these previous studies, we defined a 1-Mb candidate region (15.815–16.815 Mb on chromosome 13, GalGal6) and scrutinized it for a possible IBD signal using all available WGS data with information on the feathered-leg phenotype (supplementary table S3, Supplementary Material online). As a result, a 53-kb IBD region (chr13:16,075,704–16,128,338; fig. 3*b*) was found to be shared by three feathered-leg samples, a Beijing You individual, a Gold Brahma pool, and a Booted Bantam pool. In contrast, this region shows a homozygous wild-type pattern in three other feathered-leg samples (Pool_Langshan, a Light Sundheimer pool, and a Salmon German Faverolles pool). Furthermore, two Silkie individuals with feathered legs were both heterozygous for the 53-kb IBD haplotype which was not present in any chickens lacking feathered legs. The result shows that a second feathered-leg locus is located within this IBD region on chromosome 13 and affects the feathered-leg phenotypes in some breeds but not in others such as the Langshan used for linkage mapping in the present study. This locus is polymorphic in the Silkie breed and is thus expected to underlie phenotypic variation within this breed. Within the 53-kb IBD region, only one single nucleotide substitution, one single nucleotide deletion, and one 17.7-kb deletion were absent in all the 159 clean-leg samples. The former two sequence variants are located in intron 1 of the *H2A histone family, member Y* (*H2AFY*) gene and in sequences that are not conserved among vertebrates. The 17.7-kb deletion (chr13:16,089,993–16,107,660) involves mainly noncoding DNA, and part of a long noncoding RNA, LOC107054564, without known function. In fact, multiple evolutionary CEs are located within the deleted region (fig. 3*b*), including five which shows highly significant sequence identities to both human and mouse. According to the 77 vertebrates basewise PhyloP conservation score, only one of these CEs was annotated in human (hs1473). The nucleotide identity between partial human hs1473 and the homologous 472-bp chicken sequence (chr13:16,098,858–16,099,329) is 63% (supplementary fig. S1*b*, Supplementary Material online). Interestingly, the 17.7-kb deletion in chicken is located within the homologous region corresponding to the 44-kb deletion upstream of *PITX1* associated with feathered legs in pigeons (Domyan et al. 2016) (fig. 3*b*).

### Diagnostic Tests

Diagnostic tests for the *TBX5* SNP and the *PITX1* deletion were carried out using 295 individual samples representing 168 different chicken populations, 41 classified as feathered leg and 127 as clean leg. None of the 127 populations that are clean leg carried the *TBX5* SNP on chromosome 15 (g.12573054T>C) nor the 17.7-kb deletion on chromosome 13. All individuals from the 41 feathered-leg populations, except two Mille Fleur Brahma birds, have at least one copy of the *TBX5* mutation, whereas at least one copy of the *PITX1* deletion allele was found in birds from 25 out of the 41 feathered-leg populations (supplementary table S4, Supplementary Material online). The two Mille Fleur Brahma birds supposed to have feathered leg but which carried neither of the two mutations may carry another mutation at one of these loci or at a third leg feathering locus. However, because this was observed in a single population, we cannot exclude that a phenotype misclassification or a sample mix-up may have occurred in 1 out of 168 populations. The genotype distribution at *TBX5* and *PITX1* across breeds is summarized in table 1.

**Table 1.** Genotype Distributions in Feathered-Leg and Clean-Leg Chickens Analyzed by Whole-Genome Sequencing or a Diagnostic Test.

Breed	Genotype of *Pti-1* (*TBX5*)			Genotype of *Pti-2* (*PITX1*)		
	*Pti-1*/*Pti-1*	*Pti-1*/*pti-1^+^*	*pti-1^+^*/*pti-1^+^*	*Pti-2*/*Pti-2*	*Pti-2*/*pti-2^+^*	*pti-2^+^*/*pti-2^+^*
Feathered-leg chickens						
Beijing You	1	0	0	1	0	0
Booted Bantam	1	0	0	1	0	0
Brahama	9	2	2	4	1	8
Cochin	15	2	0	11	3	3
D’Uccle Belgiana	2	0	0	2	0	0
Faverolle	8	1	0	0	0	9
Langshan	5	0	0	0	0	5
New Hampshire × Silkie	0	2	0	0	1	1
Marans	2	4	0	0	0	6
Silkie	19	0	0	7	10	2
Sultan	5	2	0	6	1	0
Sundheimer	1	0	0	0	0	1
Clean-leg chickens^a^	0	0	379	0	0	379

a These 379 individuals lacking feathered legs represent 85 different breeds of chicken.

### Phenotypic Effects of Two Candidate Mutations

**Fig. 4.**
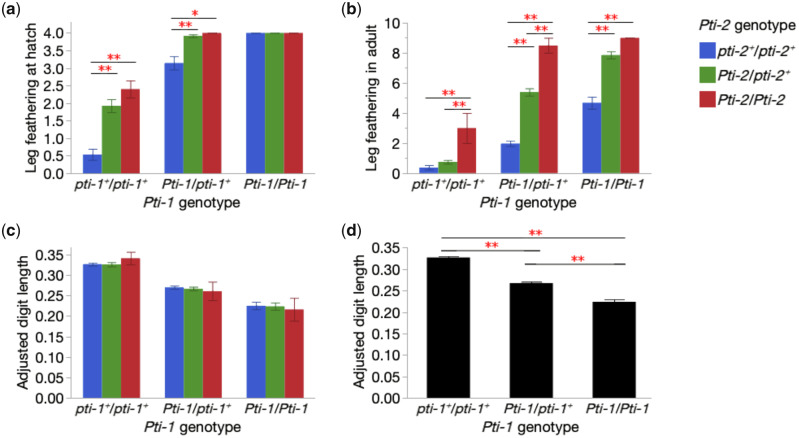
Phenotypic effects of *Pti-1* (*TBX5*) and *Pti-2* (*PITX1*) genotypes in an F_2_ intercross between Silkie (*Pti-1/Pti-1, Pti-2/Pti-2*) and Houdan (*pti-1^+^/pti-1^+^, pti-2^+^/pti-2^+^*) chicken. The error bars represent SD. Significant differences are indicated by “*” (*P* < 0.05) or “**” (*P* < 0.01). Leg feathering scores at hatch (*a*) and at 27 weeks of age (adult) (*b*) are plotted for the nine different genotype combinations. Higher scores indicate heavier feathered leg. Adjusted digit length, calculated by the length of the fourth digit divided by the length of the shank from the same individual, was plotted for the nine different genotype combinations (*c*) as well as for the *Pti-1* genotypes only (*d*).

A second mapping population consisting of an F_2_ intercross between Houdan (clean leg) and Silkie (feathered leg, carrying both the *TBX5* SNP and the *PITX1* deletion) chickens was also analyzed to further explore genotype–phenotype relationships for *TBX5* and *PITX1*. The pedigree comprised 2 Silkie males, 5 Houdan females, 14 F_1_ birds, and 194 F_2_ birds. The leg feathering score at hatch was recorded for all 194 F_2_ chicks, whereas the leg feathering score in adult birds was only recorded for 174 F_2_ chickens because of mortality. In this material, we also calculated adjusted digit length (relative to the shank length, the fourth digit only) for the 174 adult F_2_ chickens because a previous study reported that feathered leg maybe associated with brachydactyly in chicken (Lambert and Knox 1929). Nine different genotype combinations, *TBX5* (*Pti-1*) X *PITX1* (*Pti-2*), were present among the F_2_ individuals and were associated with phenotypes (fig. 4). Leg feathering phenotypes at hatch and at adult share the same trend of associations to genotypes, which is, both *Pti-1* and *Pti-2* contributes to heavier feathered leg independently and additively. In addition, each mutation shows incomplete dominance (fig. 4*a* and *b*). One exception to this general conclusion is that *Pti-1*/*Pti-1* homozygotes express the heaviest feathered leg at hatch, regardless of *Pti-2* genotype. It can be explained by the nature of down feathers, which might be suppressed to grow to become flight feather-like. Thus, the additive effects of *Pti-1* and *Pti-2* leading to even heavier feathered legs cannot be detected at hatch. Furthermore, the effect of the *Pti-2* (*PITX1*) mutation on leg feathering in adults shows a recessive inheritance in chicken that are homozygous wild-type at the *Pti-1* (*TBX5*) locus (fig. 4*b*). *Pti-2* genotypes showed no significant association to digit length (fig. 4*c*), whereas the *Pti-1* allele is significantly associated with shorter digit length, and incompletely dominant (fig. 4*d*). For both leg feathering and digit length, no gender effect was detected. Therefore, these analyses were done by pooling data from males and females.

## Discussion

The identification of causal mutations in noncoding regions of vertebrate genomes is challenging due to the difficulty in deducing or experimentally proving functional significance. Here, we have presented strong genetic evidence of causality for two noncoding mutations affecting the feathered-leg phenotype in domestic chicken. This was accomplished by combining classical pedigree analysis and linkage mapping with data mining of the extensive amount of WGS data for various populations of domestic chicken. A crucial step in this analysis was the identification of IBD regions, 30 and 53 kb, for the two loci on chromosomes 15 and 13, respectively, shared by birds showing the feathered-leg phenotype and thus expected to contain causal mutations. These rather small regions suggest a long history (hundreds of generations or more) allowing the initially large haplotype blocks associated with a causal mutation to break down due to recombination. In both cases, the genetic evidence supporting causality is convincing. The single-base change upstream of *TBX5* is in fact the only unique sequence variant within the IBD region not found on wild-type chromosomes, and out of the three unique sequence variants within the IBD region of *PITX1* the 17.7-kb deletion is the only one that disrupts evolutionary conserved sequences, and it is located within the region corresponding to the 44-kb deletion associated with feathered legs in the domestic pigeon (Domyan et al. 2016).

The results suggest that the candidate causal mutations act as *cis*-acting regulatory mutations affecting *TBX5* and *PITX1* expression. They are in fact textbook examples of such mutations because they disrupt sequences that are conserved between birds and mammals, implying functional significance. These two cases add to a growing list of regulatory mutations controlling phenotypic traits in domestic animals by altering the expression of important transcription factors. In fact, the genetic basis of leg feathering is a parallel example to the genetic basis for variation in comb morphology in chickens. The three mutations causing the three comb phenotypes Pea-comb, Rose-comb, and Duplex-comb, are all structural variants leading to ectopic expression of respectively the SOX5, MNR2, and EOMES transcription factors during comb development (Wright et al. 2009; Imsland et al. 2012; Dorshorst et al. 2015).

Our findings are in agreement with the model presented by Somes (1992) of two independent dominant mutations (*Pti-1* and *-2*) controlling leg feathering in chickens. Based on the data presented in this previous study, we can deduce that the *Pti-1* locus corresponds to the *TBX5* locus, because the Langshan breed should carry the dominant mutation at *Pti-1*, but be wild-type at *Pti-2* (*PITX1*), as reported here. Furthermore, Somes (1992) showed that the *Pti-1* mutation occurs in most feathered-leg breeds whereas the *Pti-2* mutation is restricted to the breeds with the most extensive leg feathering, in line with the distribution of the 17.7-kb deletion upstream of *PITX1* in our data (table 1). It is still an open question whether there is a third major locus affecting leg feathering in chicken. We found that 29 out of the 44 backcross individuals in our Langshan/Houdan pedigree showing intermediate leg feathering were homozygous wild-type at both *TBX5* and *PITX1*. This may therefore reflect segregation at a third locus with a modest effect on the phenotype or polygenic variation that has been selected to enhance leg feathering in the Langshan breed.

In the cross-breeding of Cochin with Leghorn as well as between Sultan and Leghorn, Somes (1992) found a 15:1 ratio between feathered and clean-leg F_2_ individuals. He proposed that two dominant loci for feathered leg segregated independently in those F_2_ populations and contributed additively to leg feathering (Somes 1992). We confirmed his results by precisely genotyping the two feathered-leg genes. We also revealed the incomplete dominance for both genes (fig. 4*a* and *b*) by recording the feather leg phenotype in more detail, rather than as either feathered or clean leg. Some of the F_2_ individuals from the Silkie/Houdan intercross showed light leg feathering despite the fact that they neither carried the *Pti-1* nor *Pti-2* mutation (fig. 4*a* and *b*). This suggests the existence of other feathered-leg loci with minor effect, awaiting to be identified.

There is a remarkable convergence as regards the evolution of the feathered-leg phenotype in domestic chicken and in domestic pigeons (Domyan et al. 2016). Firstly, the same two loci, *TBX5* and *PITX1*, are causing leg feathering in both species. Secondly, in both cases the combined effect of the two dominant mutations results in more extensive feathering. Furthermore, in pigeons the *TBX5* locus affects length of feathers whereas *PITX1* affects the location of leg feathers. This is consistent with the model for chicken presented by Somes (1992) concerning the phenotypic effects of *Pti-1* (*TBX5*) and *Pti-2* (*PITX1*). Thirdly, the morphological changes to the hindlimbs of feathered-leg pigeons also include the musculoskeletal system. Here, we showed that the length of the fourth digit (where the leg feathering is mostly found, other than shank) is shorter in chickens with the *TBX5* mutation whereas no effect due to the *PITX1* mutation was detected (fig. 4*c* and *d*). This finding is in line with the hypothesis that ectopic expression of *TBX5* in hindlimb changes its identity to be more forelimb-like. The result is consistent with a previous report that the feathered-leg gene had a pleiotropic effect on brachydactyly in chicken (Lambert and Knox 1929). Fourthly, large deletions upstream of *PITX1* are found in both pigeons and chickens, and the 17.7-kb deletion in chicken is located within a region corresponding to the 44-kb deletion in pigeons (fig. 3*b*). Our results are consistent with the proposed model for pigeons that reduced expression of the hindlimb-specific transcription factor *PITX1* and ectopic expression of the forelimb-specific transcription factor *TBX5* in hindlimb buds lead to a shift in limb identity and development of feathered legs (Domyan et al. 2016). Our identification of the causal variant at the *TBX5* locus also suggests a plausible mechanism for the previously reported ectopic *TBX5* expression in chickens with feathered legs (Domyan et al. 2016). The single-base change located 25 kb upstream of the *TBX5* coding sequence is predicted to disrupt the binding site of the caudal type homeobox 1 (CDX1) transcription factor (supplementary fig. S1*a*, Supplementary Material online) that in fact is expressed in vertebrate hindlimb buds (Gaunt et al. 2003) and which has been reported to be a repressor of *TBX5* in zebrafish embryos (Lengerke et al. 2011). Thus, we hypothesize that the disruption of the CDX1 binding site by the *TBX5* single-base change is causing ectopic expression of *TBX5* in hindlimb buds.

The 17.7-kb deletion in chickens and the 44-kb deletion in pigeons associated with the feathered-leg phenotypes overlap (fig. 3*b*) and are located ∼200 kb upstream of *PITX1*. The deleted regions harbor a number of CEs and the most prominent one is the region corresponding to the CE hs1473 in the human genome. In human, hs1473 can drive gene expression in both forelimbs and hindlimbs, and has hence been named the *pan-limb enhancer* or *Pen* (Thompson et al. 2018). However, because of different chromatin configurations, *Pen* and *PITX1* interact in hindlimbs but are physically separated in forelimbs, which results in the expression of *Pitx1* in hindlimbs but not in forelimbs (Kragesteen et al. 2018). Spielmann et al. (2012) showed that Liebenberg syndrome in human was associated with structural mutations which move *Pen* in the vicinity of *PITX1* resulting in ectopic expression of *PITX1* in forelimb and homeotic arm-to-leg transformation. Thus, the deletion of an enhancer element in chickens and pigeons is expected to cause reduced hindlimb expression of *PITX1* which was also found in pigeons with feathered legs (Domyan et al. 2016). However, they failed to find altered expression of *PITX1* in feathered-leg chickens. It is likely that the lack of altered expression of *PITX1* may have occurred because the two birds (a Silkie and a Cochin bird) used in these experiments did not carry the *PITX1* mutation since our screen showed that the mutation is not fixed in these breeds (table 1). In fact, a parallel study by Bortoluzzi et al. (2020) documented, as predicted, that *PITX1* is significantly downregulated in hindlimbs at Hamburger Hamilton stage 35 (HH35) but not at HH39 in chicken carrying the *PITX1* deletion.

The present study illustrates that novel phenotypic traits in domestic animals are often caused by the same mutations across divergent populations because favored mutations have been spread from population to population due to strong phenotypic selection (Andersson 2016). The extensive collection of WGS data now provides a powerful resource to identify IBD regions and corresponding causal mutations associated with these phenotypes as successfully accomplished in this study.

## Materials and Methods

### Animals

Procedures for this study were approved by the Institutional Animal Care and Use Committee at Virginia Tech. A linkage mapping population was initiated by crossing three Mottled Houdan males with six Black Langshan females, all purchased from Murray McMurray Hatchery (www.mcmurrayhatchery.com, Webster City, IA). Matings between 25 F_1_ females and 3 F_0_ Houdan males produced 222 backcross chickens. At 12 weeks of age, the feathered-leg phenotypes observed in the backcross population were classified into three categories: 1) clean leg, which resembled the phenotypes of the F_0_ Houdan individuals (*n* = 82); 2) intermediate feathered leg (*n* = 44); and 3) feathered leg, which resembled the phenotypes of the F_0_ Langshan birds (*n* = 96) (fig. 1).

### SNP-MaP analysis

Two DNA pools were constructed based on the phenotypes of the backcross individuals (Pool_feathered and Pool_clean). Each individual contributed 250 ng of genomic DNA to the pool. Loci controlling the feathered-leg phenotype were initially mapped by estimating SNP allele frequencies in the two DNA pools. This was accomplished using a high-density 60k SNP Illumina iSelect chicken array and the data were analyzed using the SNP-MaP (SNP Microarray and Pooling) approach (Docherty et al. 2007). The Illumina array provided an intensity reading for the two alleles (X and Y) at each of the 57,636 SNPs that were reliably scored in this material. RAF at each SNP for each DNA pool were calculated as *X*/(*X* + *Y*), where *X* and *Y* represented the intensity signals of each allele. For each SNP, absolute RAF differences (absRAFdif) were calculated by pairwise comparisons of DNA pools. The absRAFdif was then plotted against SNP genomic locations (Wells et al. 2012). The absRAFdif value of a SNP was expected to be highest in the vicinity of sequence variants affecting the feathered-leg phenotype.

### Whole-Genome Sequencing

Two DNA Pools were constructed using the parental individuals in our mapping population (Pool_Langshan and Pool_Houdan) and these were subjected to WGS together with two pools of backcross individuals (Pool_feathered and Pool_clean). The four pools were each sequenced to 30× coverage on Illumina HiSeqX with 2×150-bp paired-end reads. The data have been submitted to the SRA as BioProject ID PRJNA600264.

WGS data from Pool_Langshan and Pool_Houdan together with publicly available WGS data from 165 individuals or pooled samples were analyzed (supplementary table S3, Supplementary Material online). The latter included 7 samples from feathered-leg chickens and 158 samples from chickens with clean legs. All Illumina paired-end FASTQ data were aligned to the red junglefowl genome assembly version GalGal6 using BWA (version: 0.7.12), sorted with SAMtools (version: 1.6), and variants were called with GATK HaplotypeCaller 3.8 (Poplin et al. 2018). Structural variants were called with Lumpy (version: 0.2.13) (Layer et al. 2014). Sequence data from the backcross population (Pool_feathered and Pool_clean) were analyzed according to the same procedure, and were used to calculate pairwise F_ST_ values with Popoolation2 (Kofler et al. 2011) in a sliding window approach with window size of 50 kb and step size of 10 kb and pool sizes of 96 feathered-leg individuals: 82 clean-leg individuals. The F_ST_ values were Z-normalized and plotted against genomic locations.

### Linkage Mapping

Because of possible microarray-based error and errors when construction DNA pools, the estimated allele frequencies from SNP-MaP are arbitrary (Macgregor 2007). Individual genotyping was therefore necessary to confirm the candidate region on chromosome 15 detected using pooled SNP analysis. For the first round of linkage mapping, we selected five SNPs which had relatively high absRAFdif values within the region on chromosome 15 (supplementary table S1, Supplementary Material online) for further analysis, via the Kompetitive allele-specific PCR assay (KASP), developed by LGC Genomics (Beverly, MA; www.lgcgenomics.com) (Semagn et al. 2014). The five SNPs were genotyped using individual samples from the entire linkage mapping population except the backcross individuals with an intermediate feathered-leg phenotype. KASP assays were conducted with the mix of 2.5 μl of KASP V4.0 2× Mastermix (LGC Genomics, Beverly, MA; www.lgcgenomics.com), 1.5 μl PCR grade water, 1 μl DNA (50 ng/μl), and 0.07 μl of primers mix (12 μM each of allele-specific primer, carrying standard FAM or HEX compatible tails, and 32 μM of allele-flanking primer). Amplifications using three protocols were carried out on a Bio-Rad CFX384 Touch Real-Time PCR Detection System. PCR amplification protocol 1 (KASP-1) began with 94 °C for 15 min, 10 cycles of 94 °C for 20 s and 61 °C (−0.6 °C/cycle) for 1 min each, followed by 26 cycles of 94 °C for 20 s and 55 °C for 1 min each. KASP-2 began with 94 °C for 15 min, 10 cycles of 94 °C for 20 s and 66 °C (−0.6 °C/cycle) for 1 min each, followed by 26 cycles of 94 °C for 20 s and 57 °C for 1 min each. KASP-3 began with 94 °C for 15 min, 10 cycles of 94 °C for 20 s and 58 °C (−0.6 °C/cycle) for 1 min each, followed by 26 cycles of 94 °C for 20 s and 52 °C for 1 min each. All three protocols ended with an endpoint fluorescence reading after 37 °C for 1 min. The readings were analyzed using the Bio-Rad CFX Manager Software. Genotyping results were validated by at least two replicates for each sample. The details of these KASP assay are shown in supplementary table S1, Supplementary Material online. Linkage mapping was carried out using the CRIMAP software (Green et al. 1990) including the calculation of map distances and log10 odds ratio between individual SNPs and the feathered-leg locus on chromosome 15.

Within the candidate region defined by these five SNPs, six more SNPs, fixed for different alleles in the parental lines and identified by WGS, were selected for genotyping (supplementary table S2, Supplementary Material online). These six SNPs were used for a second round of linkage mapping, using only two recombinant backcross individuals via standard PCR and Sanger sequencing.

### TRANSFAC Analysis

Predictions of putative transcription factor binding sites were done by the MATCH program in the TRANSFAC database (Kel et al. 2003). The vertebrate database was used and only predicted binding sites with a matrix score of 1.0 were selected for further analysis.

### Diagnostic Tests of Candidate Causal Variants

A collection of 295 chicken DNA samples from 168 different populations were used to study the association between the feathered-leg phenotype and the candidate mutations associated with *TBX5* on chromosome 15 and *PITX1* on chromosome 13. Forty-one of these populations show the feathered-leg phenotype, whereas the remaining 127 have clean legs (supplementary table S4, Supplementary Material online). The SNP g.12573054T>C upstream of *TBX5* on chromosome 15 was genotyped using the same type of KASP assay (KASP-1) described earlier, whereas the 17.7-kb deletion upstream of *PITX1* on chromosome 13 was genotyped by fragment analysis following standard PCR amplifications. The following primers were used for genotyping the deletion: Pti-2_F: 5′-TGCTTTGCTTTGGTGAGTGGA-3′, pti-2+_F: 5′-TTGGCTGCTGTGTCCCTGAT-3′, and Pti-2_R: 5′-GCTGTGCTTCGGCTAATGGAG-3′, prepared as 5 μM solutions. PCR assays were conducted with the mix of 5 μl of Platinum Green Hot Start PCR Master Mix (2×) (Invitrogen), 2 μl Pti-2_F, 0.5 μl pti-2+_F, 1.5 μl Pti-2_R, and 1 μl DNA (25 ng/μl). A standard touch-down protocol was used for the amplification. Pti-2_F and Pti-2_R amplify a 977-bp fragment when the 17.7-kb deletion is present; pti-2+_F and Pti-2_R amplify a 774-bp fragment when the wild-type allele is present. The PCR products were analyzed by agarose gel electrophoresis.

### Phenotypic Analyses

We analyzed a second pedigree segregating for both the *Pti-1* (*TBX5*) and *Pti-2* (*PITX1*) loci. This pedigree was initiated by crossing two White Silkie feathered-leg males with five Mottled Houdan wild-type females. The Silkie chicken were from the pureline maintained in NC State University, US Houdan chicken were purchased from Murray McMurray Hatchery (www.mcmurrayhatchery.com, Webster City, IA). Matings between 5 F_1_ males and 9 F_1_ females produced 194 F_2_ chickens. At hatch and 27 weeks of age, the feathered-leg phenotypes of the F_2_ population were classified into 5 categories (0–4) and 10 categories (0–9), respectively. The larger number indicates heavier feathered leg, whereas 0 means clean leg. Details of each category are present in supplementary table S5, Supplementary Material online. The length of the left shank and left fourth digit was also measured at 27 weeks of age. Adjusted digit length was calculated as the ratio between the length of the fourth digit and the shank from the same individual. The entire pedigree was genotyped for the two candidate mutations as described earlier. All calculations of means, SDs, and Student’s *t-*test were conducted using the JMP software (JMP 13, 2017, www.jmp.com), the leg feathering scores were treated as numeric data. 

## Supplementary Material

Supplementary data are available at *Molecular Biology and Evolution* online.

## Supplementary Material

msaa093_Supplementary_Data

## Notes

Whole-genome sequence data generated in this study have been submitted to the SRA as BioProject ID PRJNA600264.
