# Supplementary material for: Mutations Upstream of the TBX5 and PITX1 Transcription Factor Genes Are Associated with Feathered Legs in the Domestic Chicken
Source: Mol Biol Evol. 2020 Apr 28;37(9):2477–86. doi: 10.1093/molbev/msaa093 (PMC7475036; doi:10.1093/molbev/msaa093)
Supplement: msaa093_Supplementary_Data [file msaa093_supplementary_data.zip › msaa093-Suppl_Data/TableS5.pdf]

Table S5. Leg feathering score for the F_2 population from a Houdan x Silkie intercross

At hatch		At 27 weeks	
Leg feathering score[a]	Number of individuals	Leg feathering score[a]	Number of individuals
0	21	0	23
1	11	1	30
2	22	2	26
3	25	3	11
4	115	4	13
		5	10
		6	18
		7	21
		8	9
		9	13
Total	194	Total	174

[a] The larger number indicates heavier feathered leg, while 0 means clean leg
