# Supplementary material for: Mutations Upstream of the TBX5 and PITX1 Transcription Factor Genes Are Associated with Feathered Legs in the Domestic Chicken
Source: Mol Biol Evol. 2020 Apr 28;37(9):2477–86. doi: 10.1093/molbev/msaa093 (PMC7475036; doi:10.1093/molbev/msaa093)
Supplement: msaa093_Supplementary_Data [file msaa093_supplementary_data.zip › msaa093-Suppl_Data/Supplementary_figure_legends.pdf]

Figure S1. Sequence identity between human sequences (Hg38) and chicken sequences (GalGal6) associated with the feathered leg loci on chromosome 13 and 15. (a) A 297 bp conserved element in the chicken genome (wild-type), associated with the causal mutation upstream of *TBX5* on chromosome 15, shares 58% sequence identity to human sequence. The position of the candidate SNP is marked with an arrow. The putative binding site for CDX1 is boxed. (b) A 472 bp conserved element in the chicken genome, located within the 17.7 kb deletion upstream of PITX1 on chromosome 13, shares 63% sequence identity to part of the conserved element hs1473 in the human genome and named *pan-limb enhancer* or *Pen* (Thompson et al. 2018).
