# Supplementary material for: Mutations Upstream of the TBX5 and PITX1 Transcription Factor Genes Are Associated with Feathered Legs in the Domestic Chicken
Source: Mol Biol Evol. 2020 Apr 28;37(9):2477–86. doi: 10.1093/molbev/msaa093 (PMC7475036; doi:10.1093/molbev/msaa093)
Supplement: msaa093_Supplementary_Data [file msaa093_supplementary_data.zip › msaa093-Suppl_Data/TableS1.pdf]

Table S1. First round of linkage mapping of the feathered leg locus using individual SNP analysis

dbSNP	absRAFdif[a]	Position on Chr. 15 (bp)	KASP protocol	Genetic distance from feathered-leg (cM)[b]	LOD score[b]
rs13528978	0.36	11,010,736	KASP-3	6.8	32.3
rs14096198	0.42	11,939,078	KASP-1	1.1	48.8
rs316054667	0.43	12,534,354	KASP-2	0	53.6
rs14096597	0.44	12,733,336	KASP-1	0	53.6
rs15787779	0.27	12,913,145	KASP-1	0	38.2

[a] Calculated by contrasting RAF values for feathered leg and clean leg DNA pools

[b] Calculated using the CRIMAP software, a LOD score > 3.0 is considered genome-wide significant
