# Supplementary material for: Mutations Upstream of the TBX5 and PITX1 Transcription Factor Genes Are Associated with Feathered Legs in the Domestic Chicken
Source: Mol Biol Evol. 2020 Apr 28;37(9):2477–86. doi: 10.1093/molbev/msaa093 (PMC7475036; doi:10.1093/molbev/msaa093)
Supplement: msaa093_Supplementary_Data [file msaa093_supplementary_data.zip › msaa093-Suppl_Data/TableS2.pdf]

Table S2. PCR primers for genetic markers used for the second round of linkage mapping of the feathered leg locus

dbSNP	Position on Chr. 15 (bp)	Allele in Houdan	Allele in Langshan	Primer sequence (5'-3')
rs316500191	12,167,175	G	A	F: CCTCGGCTCTTGCTCTCATAAT<br>R: GCATATTGGCAAGTGAGCAAAG
rs731374425	12,319,425	A	G	F: TGACAGGGAAAGGTGAAGTGAA<br>R: ACGTTCTGGCCCATTAGATGTT
rs738903480	12,411,252	C	T	F: AGGTTATAAATCGGGTGGCAGA<br>R: CTGGTTTCCCACTCCACCTAAC
Not reported	12,505,096	G	A	F: GTTCATGTTGTGGGTGGGAGTA<br>R: CAGCTCGACACTTTTTGCTGAT
rs732437313	12,596,017	T	C	F: GCGTGATGCCTAAAGTGTGC<br>R: TCTGTGAGCGCAGAGAGGAG
rs734977596	12,634,920	G	T	F: ACAGCCCTTGTTGGCTTTGT<br>R: TGCTGACCATCCTCTGTGCT
