# Supplementary material for: Mutations Upstream of the TBX5 and PITX1 Transcription Factor Genes Are Associated with Feathered Legs in the Domestic Chicken
Source: Mol Biol Evol. 2020 Apr 28;37(9):2477–86. doi: 10.1093/molbev/msaa093 (PMC7475036; doi:10.1093/molbev/msaa093)
Supplement: msaa093_Supplementary_Data [file msaa093_supplementary_data.zip › msaa093-Suppl_Data/TableS3.pdf]

Table S3. Public whole genome sequence data used in this study

Breed	Variety	Library Type	BioSample Accession	Genotype	
Beijing You[a]		Individual	SAMN02486156	*Pti-1*/*Pti-1*	*Pti-2*/*Pti-2*
Booted Bantam[a]	Millefleur	Pool	SAMEA104432193	*Pti-1*/*Pti-1*	*Pti-2*/*Pti-2*
Brahma[a]	Gold	Pool	SAMEA104432186	*Pti-1*/*Pti-1*	*Pti-2*/*Pti-2*
Silkie[a]	White	Pool	SAMEA104432214	*Pti-1*/*Pti-1*	Non-fixation
Silkie[a]		Individual	SAMN02142123	*Pti-1*/*Pti-1*	Non-fixation
German Faverolles[a]	Salmon	Pool	SAMEA104432192	*Pti-1*/*Pti-1*	*pti-2*$^+$/*pti-2*$^+$
Sundheimer[a]	Light	Pool	SAMEA104432216	*Pti-1*/*Pti-1*	*pti-2*$^+$/*pti-2*$^+$
Pool_Langshan[a]		Pool	SAMN13810355	*Pti-1*/*Pti-1*	*pti-2*$^+$/*pti-2*$^+$
Araucana		Individual	SAMN03940098	*pti-1*$^+$/*pti-1*$^+$	*pti-2*$^+$/*pti-2*$^+$
Araucana		Individual	SAMN03940099	*pti-1*$^+$/*pti-1*$^+$	*pti-2*$^+$/*pti-2*$^+$
Araucana		Individual	SAMN03940100	*pti-1*$^+$/*pti-1*$^+$	*pti-2*$^+$/*pti-2*$^+$
Aseel	Red Mottled	Pool	SAMEA104432184	*pti-1*$^+$/*pti-1*$^+$	*pti-2*$^+$/*pti-2*$^+$
Barbue D'anvers	Quail	Pool	SAMEA104432182	*pti-1*$^+$/*pti-1*$^+$	*pti-2*$^+$/*pti-2*$^+$
Broiler1		Individual	SAMN07327526	*pti-1*$^+$/*pti-1*$^+$	*pti-2*$^+$/*pti-2*$^+$
Broiler2		Individual	SAMN07327528	*pti-1*$^+$/*pti-1*$^+$	*pti-2*$^+$/*pti-2*$^+$
Broiler3		Individual	SAMN07327530	*pti-1*$^+$/*pti-1*$^+$	*pti-2*$^+$/*pti-2*$^+$
Broiler4		Individual	SAMN07327527	*pti-1*$^+$/*pti-1*$^+$	*pti-2*$^+$/*pti-2*$^+$
Broiler5		Individual	SAMN07327529	*pti-1*$^+$/*pti-1*$^+$	*pti-2*$^+$/*pti-2*$^+$
Broiler6		Individual	SAMN07327531	*pti-1*$^+$/*pti-1*$^+$	*pti-2*$^+$/*pti-2*$^+$
Brown Line		Individual	SAMN02941161	*pti-1*$^+$/*pti-1*$^+$	*pti-2*$^+$/*pti-2*$^+$
Burmese RJF		Pool	SAMEA104432195	*pti-1*$^+$/*pti-1*$^+$	*pti-2*$^+$/*pti-2*$^+$
Chahua		Pool	SAMN04455019	*pti-1*$^+$/*pti-1*$^+$	*pti-2*$^+$/*pti-2*$^+$
Commercial Layer	Roman	Individual	SAMN02712022	*pti-1*$^+$/*pti-1*$^+$	*pti-2*$^+$/*pti-2*$^+$
Cornish		Individual	SAMN02486157	*pti-1*$^+$/*pti-1*$^+$	*pti-2*$^+$/*pti-2*$^+$
Dongxiang		Individual	SAMN02486158	*pti-1*$^+$/*pti-1*$^+$	*pti-2*$^+$/*pti-2*$^+$
Dutch	White Crested	Pool	SAMEA104432196	*pti-1*$^+$/*pti-1*$^+$	*pti-2*$^+$/*pti-2*$^+$
East Friesian Gulls	Silver Penciled	Pool	SAMEA104432208	*pti-1*$^+$/*pti-1*$^+$	*pti-2*$^+$/*pti-2*$^+$
Fat Line		Individual	SAMN02782188	*pti-1*$^+$/*pti-1*$^+$	*pti-2*$^+$/*pti-2*$^+$
Fat Line		Individual	SAMN02782189	*pti-1*$^+$/*pti-1*$^+$	*pti-2*$^+$/*pti-2*$^+$
Fat Line		Individual	SAMN02782190	*pti-1*$^+$/*pti-1*$^+$	*pti-2*$^+$/*pti-2*$^+$
Fat Line		Individual	SAMN02782191	*pti-1*$^+$/*pti-1*$^+$	*pti-2*$^+$/*pti-2*$^+$
Fat Line		Individual	SAMN02782192	*pti-1*$^+$/*pti-1*$^+$	*pti-2*$^+$/*pti-2*$^+$
Fat Line		Individual	SAMN02782193	*pti-1*$^+$/*pti-1*$^+$	*pti-2*$^+$/*pti-2*$^+$
Fat Line		Individual	SAMN02782194	*pti-1*$^+$/*pti-1*$^+$	*pti-2*$^+$/*pti-2*$^+$
Fat Line		Individual	SAMN02782195	*pti-1*$^+$/*pti-1*$^+$	*pti-2*$^+$/*pti-2*$^+$
Fat Line		Individual	SAMN03009569	*pti-1*$^+$/*pti-1*$^+$	*pti-2*$^+$/*pti-2*$^+$
Fat Line		Individual	SAMN03009570	*pti-1*$^+$/*pti-1*$^+$	*pti-2*$^+$/*pti-2*$^+$
Fat Line		Individual	SAMN03009571	*pti-1*$^+$/*pti-1*$^+$	*pti-2*$^+$/*pti-2*$^+$
Fat Line		Individual	SAMN03009572	*pti-1*$^+$/*pti-1*$^+$	*pti-2*$^+$/*pti-2*$^+$
Fat Line		Individual	SAMN03009573	*pti-1*$^+$/*pti-1*$^+$	*pti-2*$^+$/*pti-2*$^+$
Fat Line		Individual	SAMN03009574	*pti-1*$^+$/*pti-1*$^+$	*pti-2*$^+$/*pti-2*$^+$
Fat Line		Individual	SAMN03009575	*pti-1*$^+$/*pti-1*$^+$	*pti-2*$^+$/*pti-2*$^+$
Fat Line		Individual	SAMN03009576	*pti-1*$^+$/*pti-1*$^+$	*pti-2*$^+$/*pti-2*$^+$

Breed	Variety	Library Type	BioSample Accession	Genotype	
Fayoumi		Pool	SAMN06612097	$pti\text{-}1^{+}/pti\text{-}1^{+}$	$pti\text{-}2^{+}/pti\text{-}2^{+}$
Gyeongbuk Araucana		Individual	SAMN03276499	$pti\text{-}1^{+}/pti\text{-}1^{+}$	$pti\text{-}2^{+}/pti\text{-}2^{+}$
Gyeongbuk Araucana		Individual	SAMN03276500	$pti\text{-}1^{+}/pti\text{-}1^{+}$	$pti\text{-}2^{+}/pti\text{-}2^{+}$
Gyeongbuk Araucana		Individual	SAMN03276501	$pti\text{-}1^{+}/pti\text{-}1^{+}$	$pti\text{-}2^{+}/pti\text{-}2^{+}$
Huiyang	Bearded	Pool	SAMN04364307	$pti\text{-}1^{+}/pti\text{-}1^{+}$	$pti\text{-}2^{+}/pti\text{-}2^{+}$
Indochinese RJF		Pool	SAMEA104432194	$pti\text{-}1^{+}/pti\text{-}1^{+}$	$pti\text{-}2^{+}/pti\text{-}2^{+}$
Japanese Bantam	Black Mottled	Pool	SAMEA104432190	$pti\text{-}1^{+}/pti\text{-}1^{+}$	$pti\text{-}2^{+}/pti\text{-}2^{+}$
Japanese Bantam	Black Tailed Buff	Pool	SAMEA104432188	$pti\text{-}1^{+}/pti\text{-}1^{+}$	$pti\text{-}2^{+}/pti\text{-}2^{+}$
Java	Black	Individual	SAMD00077882	$pti\text{-}1^{+}/pti\text{-}1^{+}$	$pti\text{-}2^{+}/pti\text{-}2^{+}$
Java	Black	Individual	SAMD00077883	$pti\text{-}1^{+}/pti\text{-}1^{+}$	$pti\text{-}2^{+}/pti\text{-}2^{+}$
Java	Black	Individual	SAMD00077884	$pti\text{-}1^{+}/pti\text{-}1^{+}$	$pti\text{-}2^{+}/pti\text{-}2^{+}$
Java	Black	Individual	SAMD00077885	$pti\text{-}1^{+}/pti\text{-}1^{+}$	$pti\text{-}2^{+}/pti\text{-}2^{+}$
Java	Black	Individual	SAMD00077886	$pti\text{-}1^{+}/pti\text{-}1^{+}$	$pti\text{-}2^{+}/pti\text{-}2^{+}$
Java	Black	Individual	SAMD00077887	$pti\text{-}1^{+}/pti\text{-}1^{+}$	$pti\text{-}2^{+}/pti\text{-}2^{+}$
Java	Black	Individual	SAMD00077888	$pti\text{-}1^{+}/pti\text{-}1^{+}$	$pti\text{-}2^{+}/pti\text{-}2^{+}$
Java	Black	Individual	SAMD00077889	$pti\text{-}1^{+}/pti\text{-}1^{+}$	$pti\text{-}2^{+}/pti\text{-}2^{+}$
Java	Black	Individual	SAMD00077890	$pti\text{-}1^{+}/pti\text{-}1^{+}$	$pti\text{-}2^{+}/pti\text{-}2^{+}$
Java	Black	Individual	SAMD00077891	$pti\text{-}1^{+}/pti\text{-}1^{+}$	$pti\text{-}2^{+}/pti\text{-}2^{+}$
Kedu Hitam		Individual	SAMD00077872	$pti\text{-}1^{+}/pti\text{-}1^{+}$	$pti\text{-}2^{+}/pti\text{-}2^{+}$
Kedu Hitam		Individual	SAMD00077873	$pti\text{-}1^{+}/pti\text{-}1^{+}$	$pti\text{-}2^{+}/pti\text{-}2^{+}$
Kedu Hitam		Individual	SAMD00077874	$pti\text{-}1^{+}/pti\text{-}1^{+}$	$pti\text{-}2^{+}/pti\text{-}2^{+}$
Kedu Hitam		Individual	SAMD00077875	$pti\text{-}1^{+}/pti\text{-}1^{+}$	$pti\text{-}2^{+}/pti\text{-}2^{+}$
Kedu Hitam		Individual	SAMD00077876	$pti\text{-}1^{+}/pti\text{-}1^{+}$	$pti\text{-}2^{+}/pti\text{-}2^{+}$
Kedu Hitam		Individual	SAMD00077877	$pti\text{-}1^{+}/pti\text{-}1^{+}$	$pti\text{-}2^{+}/pti\text{-}2^{+}$
Kedu Hitam		Individual	SAMD00077878	$pti\text{-}1^{+}/pti\text{-}1^{+}$	$pti\text{-}2^{+}/pti\text{-}2^{+}$
Kedu Hitam		Individual	SAMD00077879	$pti\text{-}1^{+}/pti\text{-}1^{+}$	$pti\text{-}2^{+}/pti\text{-}2^{+}$
Kedu Hitam		Individual	SAMD00077880	$pti\text{-}1^{+}/pti\text{-}1^{+}$	$pti\text{-}2^{+}/pti\text{-}2^{+}$
Kedu Hitam		Individual	SAMD00077881	$pti\text{-}1^{+}/pti\text{-}1^{+}$	$pti\text{-}2^{+}/pti\text{-}2^{+}$
Leghorn	Dark Brown	Individual	SAMN03177330	$pti\text{-}1^{+}/pti\text{-}1^{+}$	$pti\text{-}2^{+}/pti\text{-}2^{+}$
Leghorn	White	Individual	SAMD00077892	$pti\text{-}1^{+}/pti\text{-}1^{+}$	$pti\text{-}2^{+}/pti\text{-}2^{+}$
Leghorn	White	Individual	SAMD00077893	$pti\text{-}1^{+}/pti\text{-}1^{+}$	$pti\text{-}2^{+}/pti\text{-}2^{+}$
Leghorn	White	Individual	SAMD00077894	$pti\text{-}1^{+}/pti\text{-}1^{+}$	$pti\text{-}2^{+}/pti\text{-}2^{+}$
Leghorn	White	Individual	SAMEA104432172	$pti\text{-}1^{+}/pti\text{-}1^{+}$	$pti\text{-}2^{+}/pti\text{-}2^{+}$
Leghorn	White	Individual	SAMEA104432173	$pti\text{-}1^{+}/pti\text{-}1^{+}$	$pti\text{-}2^{+}/pti\text{-}2^{+}$
Leghorn	White	Individual	SAMEA104432174	$pti\text{-}1^{+}/pti\text{-}1^{+}$	$pti\text{-}2^{+}/pti\text{-}2^{+}$
Leghorn	White	Individual	SAMEA104432175	$pti\text{-}1^{+}/pti\text{-}1^{+}$	$pti\text{-}2^{+}/pti\text{-}2^{+}$
Leghorn	White	Individual	SAMEA104432176	$pti\text{-}1^{+}/pti\text{-}1^{+}$	$pti\text{-}2^{+}/pti\text{-}2^{+}$
Leghorn	White	Individual	SAMEA104432178	$pti\text{-}1^{+}/pti\text{-}1^{+}$	$pti\text{-}2^{+}/pti\text{-}2^{+}$
Leghorn	White	Individual	SAMEA104432179	$pti\text{-}1^{+}/pti\text{-}1^{+}$	$pti\text{-}2^{+}/pti\text{-}2^{+}$
Leghorn	White	Individual	SAMEA104432180	$pti\text{-}1^{+}/pti\text{-}1^{+}$	$pti\text{-}2^{+}/pti\text{-}2^{+}$
Leghorn	White	Individual	SAMEA1069020	$pti\text{-}1^{+}/pti\text{-}1^{+}$	$pti\text{-}2^{+}/pti\text{-}2^{+}$
Leghorn	White	Individual	SAMN02486166	$pti\text{-}1^{+}/pti\text{-}1^{+}$	$pti\text{-}2^{+}/pti\text{-}2^{+}$
Leghorn	White	Individual	SAMN03438107	$pti\text{-}1^{+}/pti\text{-}1^{+}$	$pti\text{-}2^{+}/pti\text{-}2^{+}$
Leghorn	White	Individual	SAMN03438108	$pti\text{-}1^{+}/pti\text{-}1^{+}$	$pti\text{-}2^{+}/pti\text{-}2^{+}$

Breed	Variety	Library Type	BioSample Accession	Genotype	
Leghorn	White	Individual	SAMN03459116	*pti-1*$^+$/*pti-1*$^+$	*pti-2*$^+$/*pti-2*$^+$
Leghorn	White	Individual	SAMN03459118	*pti-1*$^+$/*pti-1*$^+$	*pti-2*$^+$/*pti-2*$^+$
Leghorn	White	Individual	SAMN03459119	*pti-1*$^+$/*pti-1*$^+$	*pti-2*$^+$/*pti-2*$^+$
Leghorn	White	Individual	SAMN03459120	*pti-1*$^+$/*pti-1*$^+$	*pti-2*$^+$/*pti-2*$^+$
Leghorn	White	Individual	SAMN03940091	*pti-1*$^+$/*pti-1*$^+$	*pti-2*$^+$/*pti-2*$^+$
Leghorn	White	Individual	SAMN03940092	*pti-1*$^+$/*pti-1*$^+$	*pti-2*$^+$/*pti-2*$^+$
Leghorn	White	Individual	SAMN03940093	*pti-1*$^+$/*pti-1*$^+$	*pti-2*$^+$/*pti-2*$^+$
Leghorn	White	Individual	SAMN07344373	*pti-1*$^+$/*pti-1*$^+$	*pti-2*$^+$/*pti-2*$^+$
Leghorn	White	Individual	SAMN07344374	*pti-1*$^+$/*pti-1*$^+$	*pti-2*$^+$/*pti-2*$^+$
Leghorn	White	Individual	SAMN07344375	*pti-1*$^+$/*pti-1*$^+$	*pti-2*$^+$/*pti-2*$^+$
Leghorn	White	Pool	SAMN06110480	*pti-1*$^+$/*pti-1*$^+$	*pti-2*$^+$/*pti-2*$^+$
Leghorn	White	Pool	SAMN06110480	*pti-1*$^+$/*pti-1*$^+$	*pti-2*$^+$/*pti-2*$^+$
Leghorn	White	Pool	SAMN06110480	*pti-1*$^+$/*pti-1*$^+$	*pti-2*$^+$/*pti-2*$^+$
Leghorn	White	Pool	SAMN06110480	*pti-1*$^+$/*pti-1*$^+$	*pti-2*$^+$/*pti-2*$^+$
Leghorn		Individual	SAMN03276493	*pti-1*$^+$/*pti-1*$^+$	*pti-2*$^+$/*pti-2*$^+$
Leghorn		Individual	SAMN03276494	*pti-1*$^+$/*pti-1*$^+$	*pti-2*$^+$/*pti-2*$^+$
Leghorn		Individual	SAMN03276495	*pti-1*$^+$/*pti-1*$^+$	*pti-2*$^+$/*pti-2*$^+$
Leghorn		Pool	SAMN06612098	*pti-1*$^+$/*pti-1*$^+$	*pti-2*$^+$/*pti-2*$^+$
Lhasa	White	Pool	SAMN04455031	*pti-1*$^+$/*pti-1*$^+$	*pti-2*$^+$/*pti-2*$^+$
Lhasa	White	Pool	SAMN04455035	*pti-1*$^+$/*pti-1*$^+$	*pti-2*$^+$/*pti-2*$^+$
Lhasa	White	Pool	SAMN04455079	*pti-1*$^+$/*pti-1*$^+$	*pti-2*$^+$/*pti-2*$^+$
Lhasa	White	Pool	SAMN04455095	*pti-1*$^+$/*pti-1*$^+$	*pti-2*$^+$/*pti-2*$^+$
Line A		Pool	SAMN04364308	*pti-1*$^+$/*pti-1*$^+$	*pti-2*$^+$/*pti-2*$^+$
Luxi Game		Individual	SAMN02486159	*pti-1*$^+$/*pti-1*$^+$	*pti-2*$^+$/*pti-2*$^+$
Orpington	Buff	Pool	SAMEA104432209	*pti-1*$^+$/*pti-1*$^+$	*pti-2*$^+$/*pti-2*$^+$
Plymouth Rock	White	Individual	SAMN02486167	*pti-1*$^+$/*pti-1*$^+$	*pti-2*$^+$/*pti-2*$^+$
Polish	White Crested Black	Individual	SAMN03177328	*pti-1*$^+$/*pti-1*$^+$	*pti-2*$^+$/*pti-2*$^+$
Polish		Pool	SAMEA104432210	*pti-1*$^+$/*pti-1*$^+$	*pti-2*$^+$/*pti-2*$^+$
Red Junglefowl	Java	Individual	SAMD00077852	*pti-1*$^+$/*pti-1*$^+$	*pti-2*$^+$/*pti-2*$^+$
Red Junglefowl	Java	Individual	SAMD00077853	*pti-1*$^+$/*pti-1*$^+$	*pti-2*$^+$/*pti-2*$^+$
Red Junglefowl	Java	Individual	SAMD00077854	*pti-1*$^+$/*pti-1*$^+$	*pti-2*$^+$/*pti-2*$^+$
Red Junglefowl	Sumatra	Individual	SAMD00077855	*pti-1*$^+$/*pti-1*$^+$	*pti-2*$^+$/*pti-2*$^+$
Red Junglefowl	Sumatra	Individual	SAMD00077856	*pti-1*$^+$/*pti-1*$^+$	*pti-2*$^+$/*pti-2*$^+$
Red Junglefowl		Individual	SAMN02333832	*pti-1*$^+$/*pti-1*$^+$	*pti-2*$^+$/*pti-2*$^+$
Red Junglefowl		Individual	SAMN02333833	*pti-1*$^+$/*pti-1*$^+$	*pti-2*$^+$/*pti-2*$^+$
Red Junglefowl		Individual	SAMN02486161	*pti-1*$^+$/*pti-1*$^+$	*pti-2*$^+$/*pti-2*$^+$
Red Junglefowl		Individual	SAMN02712039	*pti-1*$^+$/*pti-1*$^+$	*pti-2*$^+$/*pti-2*$^+$
Red Junglefowl		Individual	SAMN02712040	*pti-1*$^+$/*pti-1*$^+$	*pti-2*$^+$/*pti-2*$^+$
Red Junglefowl		Individual	SAMN02712041	*pti-1*$^+$/*pti-1*$^+$	*pti-2*$^+$/*pti-2*$^+$
Red Junglefowl		Individual	SAMN02712042	*pti-1*$^+$/*pti-1*$^+$	*pti-2*$^+$/*pti-2*$^+$
Red Junglefowl		Individual	SAMN02712043	*pti-1*$^+$/*pti-1*$^+$	*pti-2*$^+$/*pti-2*$^+$
REL Line		Individual	SAMN07312781	*pti-1*$^+$/*pti-1*$^+$	*pti-2*$^+$/*pti-2*$^+$
REL Line		Individual	SAMN07327525	*pti-1*$^+$/*pti-1*$^+$	*pti-2*$^+$/*pti-2*$^+$
Rhode Island	Red	Individual	SAMN02486160	*pti-1*$^+$/*pti-1*$^+$	*pti-2*$^+$/*pti-2*$^+$

Breed	Variety	Library Type	BioSample Accession	Genotype	
Rhode Island	Red	Pool	SAMEA104432211	*pti-1*$^+$/*pti-1*$^+$	*pti-2*$^+$/*pti-2*$^+$
Rhode Island	White	Individual	SAMN02444485	*pti-1*$^+$/*pti-1*$^+$	*pti-2*$^+$/*pti-2*$^+$
Rhode Island	White	Individual	SAMN02444503	*pti-1*$^+$/*pti-1*$^+$	*pti-2*$^+$/*pti-2*$^+$
Rose Comb Bantam	Black	Pool	SAMEA104432185	*pti-1*$^+$/*pti-1*$^+$	*pti-2*$^+$/*pti-2*$^+$
Rumpless Araucana	Black	Pool	SAMEA104432183	*pti-1*$^+$/*pti-1*$^+$	*pti-2*$^+$/*pti-2*$^+$
Sebright Bantam	Silver	Pool	SAMEA104432213	*pti-1*$^+$/*pti-1*$^+$	*pti-2*$^+$/*pti-2*$^+$
Shamo	Black	Pool	SAMEA104432215	*pti-1*$^+$/*pti-1*$^+$	*pti-2*$^+$/*pti-2*$^+$
Shouguang		Individual	SAMN02486162	*pti-1*$^+$/*pti-1*$^+$	*pti-2*$^+$/*pti-2*$^+$
Sicilian Buttercup		Individual	SAMN03177329	*pti-1*$^+$/*pti-1*$^+$	*pti-2*$^+$/*pti-2*$^+$
Smyth Line		Individual	SAMN02941162	*pti-1*$^+$/*pti-1*$^+$	*pti-2*$^+$/*pti-2*$^+$
Sumatra	Black	Individual	SAMD00077862	*pti-1*$^+$/*pti-1*$^+$	*pti-2*$^+$/*pti-2*$^+$
Sumatra	Black	Individual	SAMD00077863	*pti-1*$^+$/*pti-1*$^+$	*pti-2*$^+$/*pti-2*$^+$
Sumatra	Black	Individual	SAMD00077864	*pti-1*$^+$/*pti-1*$^+$	*pti-2*$^+$/*pti-2*$^+$
Sumatra	Black	Individual	SAMD00077865	*pti-1*$^+$/*pti-1*$^+$	*pti-2*$^+$/*pti-2*$^+$
Sumatra	Black	Individual	SAMD00077866	*pti-1*$^+$/*pti-1*$^+$	*pti-2*$^+$/*pti-2*$^+$
Sumatra	Black	Individual	SAMD00077867	*pti-1*$^+$/*pti-1*$^+$	*pti-2*$^+$/*pti-2*$^+$
Sumatra	Black	Individual	SAMD00077868	*pti-1*$^+$/*pti-1*$^+$	*pti-2*$^+$/*pti-2*$^+$
Sumatra	Black	Individual	SAMD00077869	*pti-1*$^+$/*pti-1*$^+$	*pti-2*$^+$/*pti-2*$^+$
Sumatra	Black	Individual	SAMD00077870	*pti-1*$^+$/*pti-1*$^+$	*pti-2*$^+$/*pti-2*$^+$
Sumatra	Black	Individual	SAMD00077871	*pti-1*$^+$/*pti-1*$^+$	*pti-2*$^+$/*pti-2*$^+$
Sumatra	Black	Pool	SAMEA104432212	*pti-1*$^+$/*pti-1*$^+$	*pti-2*$^+$/*pti-2*$^+$
Sumatra		Individual	SAMD00077857	*pti-1*$^+$/*pti-1*$^+$	*pti-2*$^+$/*pti-2*$^+$
Sumatra		Individual	SAMD00077858	*pti-1*$^+$/*pti-1*$^+$	*pti-2*$^+$/*pti-2*$^+$
Sumatra		Individual	SAMD00077859	*pti-1*$^+$/*pti-1*$^+$	*pti-2*$^+$/*pti-2*$^+$
Sumatra		Individual	SAMD00077860	*pti-1*$^+$/*pti-1*$^+$	*pti-2*$^+$/*pti-2*$^+$
Sumatra		Individual	SAMD00077861	*pti-1*$^+$/*pti-1*$^+$	*pti-2*$^+$/*pti-2*$^+$
Pool_Houdan		Pool	SAMN13810356	*pti-1*$^+$/*pti-1*$^+$	*pti-2*$^+$/*pti-2*$^+$
Wenchang		Individual	SAMN02486165	*pti-1*$^+$/*pti-1*$^+$	*pti-2*$^+$/*pti-2*$^+$
Xishuangbanna Game		Individual	SAMN02712023	*pti-1*$^+$/*pti-1*$^+$	*pti-2*$^+$/*pti-2*$^+$
Xishuangbanna Game		Individual	SAMN02712024	*pti-1*$^+$/*pti-1*$^+$	*pti-2*$^+$/*pti-2*$^+$
Xishuangbanna Game		Individual	SAMN02712025	*pti-1*$^+$/*pti-1*$^+$	*pti-2*$^+$/*pti-2*$^+$
Xishuangbanna Game		Individual	SAMN02712026	*pti-1*$^+$/*pti-1*$^+$	*pti-2*$^+$/*pti-2*$^+$
Xishuangbanna Game		Individual	SAMN02712027	*pti-1*$^+$/*pti-1*$^+$	*pti-2*$^+$/*pti-2*$^+$
Xishuangbanna Game		Individual	SAMN02712028	*pti-1*$^+$/*pti-1*$^+$	*pti-2*$^+$/*pti-2*$^+$
Xishuangbanna Game		Individual	SAMN02712029	*pti-1*$^+$/*pti-1*$^+$	*pti-2*$^+$/*pti-2*$^+$
Xishuangbanna Game		Individual	SAMN02712030	*pti-1*$^+$/*pti-1*$^+$	*pti-2*$^+$/*pti-2*$^+$

[a] Chicken samples with feathered leg phenotype. Others express clean leg
