# Supplementary material for: Mutations Upstream of the TBX5 and PITX1 Transcription Factor Genes Are Associated with Feathered Legs in the Domestic Chicken
Source: Mol Biol Evol. 2020 Apr 28;37(9):2477–86. doi: 10.1093/molbev/msaa093 (PMC7475036; doi:10.1093/molbev/msaa093)
Supplement: msaa093_Supplementary_Data [file msaa093_supplementary_data.zip › msaa093-Suppl_Data/TableS4.pdf]

Table S4. List of DNA samples used for diagnostic tests for *Pti-1* (*TBX5*) and *Pti-2 (PITX1)*

Breed	Variety	Source	Number of animals	Genotype	
A (B^{Sd})	Sex-linked Dilution	Tucson, AZ, U.S.	2	*pti-1*$^{+}$/*pti-1*$^{+}$	*pti-2*$^{+}$/*pti-2*$^{+}$
Ameraucana	Black	Lucasville, OH, U.S.	2	*pti-1*$^{+}$/*pti-1*$^{+}$	*pti-2*$^{+}$/*pti-2*$^{+}$
Ameraucana	Blue	Lucasville, OH, U.S.	2	*pti-1*$^{+}$/*pti-1*$^{+}$	*pti-2*$^{+}$/*pti-2*$^{+}$
Ameraucana	Uncharacterized	Cary, NC, U.S.	1	*pti-1*$^{+}$/*pti-1*$^{+}$	*pti-2*$^{+}$/*pti-2*$^{+}$
American Longtail	Wild-type	H & H Longtails	2	*pti-1*$^{+}$/*pti-1*$^{+}$	*pti-2*$^{+}$/*pti-2*$^{+}$
Amroks Cuckoo	Uncharacterized	Canterbury, U.K.	2	*pti-1*$^{+}$/*pti-1*$^{+}$	*pti-2*$^{+}$/*pti-2*$^{+}$
Andalusian	Blue	Ideal Poultry	1	*pti-1*$^{+}$/*pti-1*$^{+}$	*pti-2*$^{+}$/*pti-2*$^{+}$
Andalusian	Blue	Marshfield, WI, U.S.	1	*pti-1*$^{+}$/*pti-1*$^{+}$	*pti-2*$^{+}$/*pti-2*$^{+}$
Andalusian	Blue	Canterbury, U.K.	2	*pti-1*$^{+}$/*pti-1*$^{+}$	*pti-2*$^{+}$/*pti-2*$^{+}$
Araucana	Uncharacterized	Macclesfield, NC, U.S.	2	*pti-1*$^{+}$/*pti-1*$^{+}$	*pti-2*$^{+}$/*pti-2*$^{+}$
Aseel	Dark	Ideal Poultry	1	*pti-1*$^{+}$/*pti-1*$^{+}$	*pti-2*$^{+}$/*pti-2*$^{+}$
Australorp	Black	Marshfield, WI, U.S.	1	*pti-1*$^{+}$/*pti-1*$^{+}$	*pti-2*$^{+}$/*pti-2*$^{+}$
Australorp	Black	Canterbury, U.K.	2	*pti-1*$^{+}$/*pti-1*$^{+}$	*pti-2*$^{+}$/*pti-2*$^{+}$
Australorp	Black Speckled	Canterbury, U.K.	2	*pti-1*$^{+}$/*pti-1*$^{+}$	*pti-2*$^{+}$/*pti-2*$^{+}$
Ayam Cemani	Black	Philippines	2	*pti-1*$^{+}$/*pti-1*$^{+}$	*pti-2*$^{+}$/*pti-2*$^{+}$
B (Wild-type)	Wild-type	Tucson, AZ, U.S.	2	*pti-1*$^{+}$/*pti-1*$^{+}$	*pti-2*$^{+}$/*pti-2*$^{+}$
Blue Egg	Uncharacterized	Tucson, AZ, U.S.	2	*pti-1*$^{+}$/*pti-1*$^{+}$	*pti-2*$^{+}$/*pti-2*$^{+}$
Brahma[a]	Buff	Macclesfield, NC, U.S.	2	*Pti-1*/*Pti-1*	*pti-2*$^{+}$/*pti-2*$^{+}$
Brahma[a]	Dark	Ideal Poultry.	1	*Pti-1*/*Pti-1*	Non-fixation
Brahma[a]	Dark	Lucasville, OH, U.S.	1	Non-fixation	*pti-2*$^{+}$/*pti-2*$^{+}$
Brahma[a]	Light	Canterbury, U.K.	2	Non-fixation	Non-fixation
Brahma[a]	Light	Raleigh, NC, U.S.	2	*Pti-1*/*Pti-1*	*pti-2*$^{+}$/*pti-2*$^{+}$
Brahma[a]	Buff	Canterbury, U.K.	2	*Pti-1*/*Pti-1*	*Pti-2*/*Pti-2*
Brahma[a]	Mille Fleur	Canterbury, U.K.	2	*pti-1*$^{+}$/*pti-1*$^{+}$	*pti-2*$^{+}$/*pti-2*$^{+}$
Buckeye	Uncharacterized	Lucasville, OH, U.S.	1	*pti-1*$^{+}$/*pti-1*$^{+}$	*pti-2*$^{+}$/*pti-2*$^{+}$
Buttercup	Buttercup	Lucasville, OH, U.S.	2	*pti-1*$^{+}$/*pti-1*$^{+}$	*pti-2*$^{+}$/*pti-2*$^{+}$
Buttercup	Buttercup	Murray McMurray	2	*pti-1*$^{+}$/*pti-1*$^{+}$	*pti-2*$^{+}$/*pti-2*$^{+}$
C (Black)	Black	Tucson, AZ, U.S.	2	*pti-1*$^{+}$/*pti-1*$^{+}$	*pti-2*$^{+}$/*pti-2*$^{+}$
Campine	Golden	Raleigh, NC, U.S.	2	*pti-1*$^{+}$/*pti-1*$^{+}$	*pti-2*$^{+}$/*pti-2*$^{+}$
Campine	Silver Pencilled	Canterbury, U.K.	2	*pti-1*$^{+}$/*pti-1*$^{+}$	*pti-2*$^{+}$/*pti-2*$^{+}$
Chantecler	Partridge	Lucasville, OH, U.S.	1	*pti-1*$^{+}$/*pti-1*$^{+}$	*pti-2*$^{+}$/*pti-2*$^{+}$
Cochin[a]	Black	Canterbury, U.K.	2	*Pti-1*/*Pti-1*	*Pti-2*/*Pti-2*
Cochin[a]	Black speckled	Canterbury, U.K.	2	*Pti-1*/*Pti-1*	*Pti-2*/*Pti-2*
Cochin[a]	Blue	Ideal Poultry	1	*Pti-1*/*Pti-1*	*Pti-2*/*Pti-2*
Cochin[a]	Blue	Ideal Poultry	2	*Pti-1*/*Pti-1*	Non-fixation
Cochin[a]	Blue	Canterbury, U.K.	2	Non-fixation	*Pti-2*/*Pti-2*
Cochin[a]	Buff	Macclesfield, NC, U.S.	1	*Pti-1*/*Pti-1*	*pti-2*$^{+}$/*pti-2*$^{+}$
Cochin[a]	Golden laced	Lucasville, OH, U.S.	1	*Pti-1*/*Pti-1*	Non-fixation
Cochin[a]	Partridge	Murray McMurray	2	*Pti-1*/*Pti-1*	*pti-2*$^{+}$/*pti-2*$^{+}$
Cochin[a]	White	Canterbury, U.K.	2	*Pti-1*/*Pti-1*	*Pti-2*/*Pti-2*
Cochin[a]	Black	Canterbury, U.K.	2	*Pti-1*/*Pti-1*	*Pti-2*/*Pti-2*
Cornish	Dark	Cackle Hatchery	1	*pti-1*$^{+}$/*pti-1*$^{+}$	*pti-2*$^{+}$/*pti-2*$^{+}$
Cornish	Dark	Murray McMurray	6	*pti-1*$^{+}$/*pti-1*$^{+}$	*pti-2*$^{+}$/*pti-2*$^{+}$

Breed	Variety	Source	Number of animals	Genotype
Cornish	Uncharacterized	Ideal Poultry	1	$pti\text{-}1^{+}/pti\text{-}1^{+}$ $pti\text{-}2^{+}/pti\text{-}2^{+}$
Crevecoeur	Uncharacterized	Ideal Poultry	1	$pti\text{-}1^{+}/pti\text{-}1^{+}$ $pti\text{-}2^{+}/pti\text{-}2^{+}$
Czech	Golden	Canterbury, U.K.	2	$pti\text{-}1^{+}/pti\text{-}1^{+}$ $pti\text{-}2^{+}/pti\text{-}2^{+}$
D (Splash)	Splash	Tucson, AZ, U.S.	2	$pti\text{-}1^{+}/pti\text{-}1^{+}$ $pti\text{-}2^{+}/pti\text{-}2^{+}$
D'Uccle Belgian[a]	Mille Fleur	Lucasville, OH, U.S.	2	*Pti-1/Pti-1* *Pti-2/Pti-2*
Delaware	Uncharacterized	Lucasville, OH, U.S.	1	$pti\text{-}1^{+}/pti\text{-}1^{+}$ $pti\text{-}2^{+}/pti\text{-}2^{+}$
Dominique	Uncharacterized	Cackle Hatchery	1	$pti\text{-}1^{+}/pti\text{-}1^{+}$ $pti\text{-}2^{+}/pti\text{-}2^{+}$
Dorking	Silver Gray	East Lansing, MI, U.S.	2	$pti\text{-}1^{+}/pti\text{-}1^{+}$ $pti\text{-}2^{+}/pti\text{-}2^{+}$
Dorking	Silver gray	Murray McMurray	2	$pti\text{-}1^{+}/pti\text{-}1^{+}$ $pti\text{-}2^{+}/pti\text{-}2^{+}$
Egyptian Fayoumin	Barred	East Lansing, MI, U.S.	1	$pti\text{-}1^{+}/pti\text{-}1^{+}$ $pti\text{-}2^{+}/pti\text{-}2^{+}$
Egyptian Fayoumin	Barred	Ideal Poultry	2	$pti\text{-}1^{+}/pti\text{-}1^{+}$ $pti\text{-}2^{+}/pti\text{-}2^{+}$
Faverolle[a]	Salmon	Canterbury, U.K.	2	Non-fixation $pti\text{-}2^{+}/pti\text{-}2^{+}$
Faverolle[a]	Salmon	Murray McMurray	2	*Pti-1/Pti-1* $pti\text{-}2^{+}/pti\text{-}2^{+}$
Faverolle[a]	Uncharacterized	Raleigh, NC, U.S.	2	*Pti-1/Pti-1* $pti\text{-}2^{+}/pti\text{-}2^{+}$
Faverolle[a]	White	Lucasville, OH, U.S.	2	*Pti-1/Pti-1* $pti\text{-}2^{+}/pti\text{-}2^{+}$
Fayoumi	Barred	Ames, IA, U.S.	2	$pti\text{-}1^{+}/pti\text{-}1^{+}$ $pti\text{-}2^{+}/pti\text{-}2^{+}$
Fayoumi	Barred	Ames, IA, U.S.	2	$pti\text{-}1^{+}/pti\text{-}1^{+}$ $pti\text{-}2^{+}/pti\text{-}2^{+}$
Hamburg	Golden Penciled	Murray McMurray	2	$pti\text{-}1^{+}/pti\text{-}1^{+}$ $pti\text{-}2^{+}/pti\text{-}2^{+}$
Hamburg	Silver Spangled	Cackle Hatchery	1	$pti\text{-}1^{+}/pti\text{-}1^{+}$ $pti\text{-}2^{+}/pti\text{-}2^{+}$
Hamburg	Silver Spangled	Macclesfield, NC, U.S.	2	$pti\text{-}1^{+}/pti\text{-}1^{+}$ $pti\text{-}2^{+}/pti\text{-}2^{+}$
Hamburg	Silver Spangled	Murray McMurray	2	$pti\text{-}1^{+}/pti\text{-}1^{+}$ $pti\text{-}2^{+}/pti\text{-}2^{+}$
Hamburg	Uncharacterized	Ideal Poultry	2	$pti\text{-}1^{+}/pti\text{-}1^{+}$ $pti\text{-}2^{+}/pti\text{-}2^{+}$
Hamburg	Silver Spangled	Canterbury, U.K.	2	$pti\text{-}1^{+}/pti\text{-}1^{+}$ $pti\text{-}2^{+}/pti\text{-}2^{+}$
Hedemora	Uncharacterized	Uppsala, Sweden	2	$pti\text{-}1^{+}/pti\text{-}1^{+}$ $pti\text{-}2^{+}/pti\text{-}2^{+}$
Houdan	Mottled	Ideal Poultry	2	$pti\text{-}1^{+}/pti\text{-}1^{+}$ $pti\text{-}2^{+}/pti\text{-}2^{+}$
Houdan	Mottled	Lucasville, OH, U.S.	1	$pti\text{-}1^{+}/pti\text{-}1^{+}$ $pti\text{-}2^{+}/pti\text{-}2^{+}$
Houdan	Mottled	Murray McMurray	2	$pti\text{-}1^{+}/pti\text{-}1^{+}$ $pti\text{-}2^{+}/pti\text{-}2^{+}$
Houdan	Mottled	Murray McMurray	2	$pti\text{-}1^{+}/pti\text{-}1^{+}$ $pti\text{-}2^{+}/pti\text{-}2^{+}$
Japanese Bantam	Uncharacterized	East Lansing, MI, U.S.	1	$pti\text{-}1^{+}/pti\text{-}1^{+}$ $pti\text{-}2^{+}/pti\text{-}2^{+}$
Junglefowl	Red	Richardson line	2	$pti\text{-}1^{+}/pti\text{-}1^{+}$ $pti\text{-}2^{+}/pti\text{-}2^{+}$
Junglefowl	Red	Raleigh, NC, U.S.	2	$pti\text{-}1^{+}/pti\text{-}1^{+}$ $pti\text{-}2^{+}/pti\text{-}2^{+}$
Langshan[a]	Black	Lucasville, OH, U.S.	2	*Pti-1/Pti-1* $pti\text{-}2^{+}/pti\text{-}2^{+}$
Langshan[a]	Black	Murray McMurray	2	*Pti-1/Pti-1* $pti\text{-}2^{+}/pti\text{-}2^{+}$
Leghorn	Black tail, red	Lucasville, OH, U.S.	2	$pti\text{-}1^{+}/pti\text{-}1^{+}$ $pti\text{-}2^{+}/pti\text{-}2^{+}$
Leghorn	Brown (Italian Partridge)	Canterbury, U.K.	2	$pti\text{-}1^{+}/pti\text{-}1^{+}$ $pti\text{-}2^{+}/pti\text{-}2^{+}$
Leghorn	Dark brown	Raleigh, NC, U.S.	1	$pti\text{-}1^{+}/pti\text{-}1^{+}$ $pti\text{-}2^{+}/pti\text{-}2^{+}$
Leghorn	Light brown	Lucasville, OH, U.S.	1	$pti\text{-}1^{+}/pti\text{-}1^{+}$ $pti\text{-}2^{+}/pti\text{-}2^{+}$
Leghorn	Light brown	Raleigh, NC, U.S.	1	$pti\text{-}1^{+}/pti\text{-}1^{+}$ $pti\text{-}2^{+}/pti\text{-}2^{+}$
Leghorn	White	Ames, IA, U.S.	2	$pti\text{-}1^{+}/pti\text{-}1^{+}$ $pti\text{-}2^{+}/pti\text{-}2^{+}$
Leghorn	White	Ames, IA, U.S.	2	$pti\text{-}1^{+}/pti\text{-}1^{+}$ $pti\text{-}2^{+}/pti\text{-}2^{+}$
Leghorn	White	Ames, IA, U.S.	2	$pti\text{-}1^{+}/pti\text{-}1^{+}$ $pti\text{-}2^{+}/pti\text{-}2^{+}$
Leghorn	White	Ideal Poultry	2	$pti\text{-}1^{+}/pti\text{-}1^{+}$ $pti\text{-}2^{+}/pti\text{-}2^{+}$
Line O	Uncharacterized	Ames, IA, U.S.	1	$pti\text{-}1^{+}/pti\text{-}1^{+}$ $pti\text{-}2^{+}/pti\text{-}2^{+}$
Line15Is	Uncharacterized	Ames, IA, U.S.	1	$pti\text{-}1^{+}/pti\text{-}1^{+}$ $pti\text{-}2^{+}/pti\text{-}2^{+}$

Breed	Variety	Source	Number of animals	Genotype	
Madagascar Game	Uncharacterized	Ideal Poultry	1	*pti-1*$^{+}$/*pti-1*$^{+}$	*pti-2*$^{+}$/*pti-2*$^{+}$
Malay	Red	Ideal Poultry	1	*pti-1*$^{+}$/*pti-1*$^{+}$	*pti-2*$^{+}$/*pti-2*$^{+}$
Marans[a]	Birchen	GreenFire Farms	2	Non-fixation	*pti-2*$^{+}$/*pti-2*$^{+}$
Marans[a]	Copper	Lucasville, OH, U.S.	2	Non-fixation	*pti-2*$^{+}$/*pti-2*$^{+}$
Marans[a]	Wheaten	Lucasville, OH, U.S.	2	Non-fixation	*pti-2*$^{+}$/*pti-2*$^{+}$
Modern Games	Black Breasted Red	Cackle Hatchery	1	*pti-1*$^{+}$/*pti-1*$^{+}$	*pti-2*$^{+}$/*pti-2*$^{+}$
Moscow Game	Uncharacterized	Canterbury, U.K.	2	*pti-1*$^{+}$/*pti-1*$^{+}$	*pti-2*$^{+}$/*pti-2*$^{+}$
New Hampshire	Red	Ideal Poultry	1	*pti-1*$^{+}$/*pti-1*$^{+}$	*pti-2*$^{+}$/*pti-2*$^{+}$
New Hampshire	Uncharacterized	Lucasville, OH, U.S.	1	*pti-1*$^{+}$/*pti-1*$^{+}$	*pti-2*$^{+}$/*pti-2*$^{+}$
New Hampshire	Uncharacterized	Canterbury, U.K.	2	*pti-1*$^{+}$/*pti-1*$^{+}$	*pti-2*$^{+}$/*pti-2*$^{+}$
New Hampshire × Silkie[a]	Uncharacterized	Raleigh, NC, U.S.	2	Non-fixation	Non-fixation
Old English	Spangled	Lucasville, OH, U.S.	2	*pti-1*$^{+}$/*pti-1*$^{+}$	*pti-2*$^{+}$/*pti-2*$^{+}$
Orloff	Speckled	Ideal Poultry	1	*pti-1*$^{+}$/*pti-1*$^{+}$	*pti-2*$^{+}$/*pti-2*$^{+}$
Orpington	Black	Marshfield, WI, U.S.	1	*pti-1*$^{+}$/*pti-1*$^{+}$	*pti-2*$^{+}$/*pti-2*$^{+}$
Orpington	Buff	Lucasville, OH, U.S.	2	*pti-1*$^{+}$/*pti-1*$^{+}$	*pti-2*$^{+}$/*pti-2*$^{+}$
Orpington	Buff	Macclesfield, NC, U.S.	1	*pti-1*$^{+}$/*pti-1*$^{+}$	*pti-2*$^{+}$/*pti-2*$^{+}$
Phoenix	Golden	Ideal Poultry	1	*pti-1*$^{+}$/*pti-1*$^{+}$	*pti-2*$^{+}$/*pti-2*$^{+}$
Phoenix	Silver	Marshfield, WI, U.S.	1	*pti-1*$^{+}$/*pti-1*$^{+}$	*pti-2*$^{+}$/*pti-2*$^{+}$
Phoenix	Silver Duckwing	Ideal Poultry	1	*pti-1*$^{+}$/*pti-1*$^{+}$	*pti-2*$^{+}$/*pti-2*$^{+}$
Plymouth Rock	Barred	Raleigh, NC, U.S.	2	*pti-1*$^{+}$/*pti-1*$^{+}$	*pti-2*$^{+}$/*pti-2*$^{+}$
Plymouth Rock	Barred	Raleigh, NC, U.S.	1	*pti-1*$^{+}$/*pti-1*$^{+}$	*pti-2*$^{+}$/*pti-2*$^{+}$
Plymouth Rock	Barred	Virginia Tech	2	*pti-1*$^{+}$/*pti-1*$^{+}$	*pti-2*$^{+}$/*pti-2*$^{+}$
Plymouth Rock	Partridge	Murray McMurray	2	*pti-1*$^{+}$/*pti-1*$^{+}$	*pti-2*$^{+}$/*pti-2*$^{+}$
Plymouth Rock	Silver pencilled	Murray McMurray	2	*pti-1*$^{+}$/*pti-1*$^{+}$	*pti-2*$^{+}$/*pti-2*$^{+}$
Polish	Buff laced	Macclesfield, NC, U.S.	2	*pti-1*$^{+}$/*pti-1*$^{+}$	*pti-2*$^{+}$/*pti-2*$^{+}$
Polish	White	Lucasville, OH, U.S.	1	*pti-1*$^{+}$/*pti-1*$^{+}$	*pti-2*$^{+}$/*pti-2*$^{+}$
Polish	White Crested Black	Murray McMurray	1	*pti-1*$^{+}$/*pti-1*$^{+}$	*pti-2*$^{+}$/*pti-2*$^{+}$
Polish	White Crested Black	Canterbury, U.K.	2	*pti-1*$^{+}$/*pti-1*$^{+}$	*pti-2*$^{+}$/*pti-2*$^{+}$
Polish	White crested Blue	Lucasville, OH, U.S.	1	*pti-1*$^{+}$/*pti-1*$^{+}$	*pti-2*$^{+}$/*pti-2*$^{+}$
Polish	White crested, black	Lucasville, OH, U.S.	1	*pti-1*$^{+}$/*pti-1*$^{+}$	*pti-2*$^{+}$/*pti-2*$^{+}$
Polish	White crested, black	Marshfield, WI, U.S.	1	*pti-1*$^{+}$/*pti-1*$^{+}$	*pti-2*$^{+}$/*pti-2*$^{+}$
Polish	White crested, black	Raleigh, NC, U.S.	2	*pti-1*$^{+}$/*pti-1*$^{+}$	*pti-2*$^{+}$/*pti-2*$^{+}$
Rhode Island	Red	East Lansing, MI, U.S.	1	*pti-1*$^{+}$/*pti-1*$^{+}$	*pti-2*$^{+}$/*pti-2*$^{+}$
Rhode Island	Red	Lucasville, OH, U.S.	1	*pti-1*$^{+}$/*pti-1*$^{+}$	*pti-2*$^{+}$/*pti-2*$^{+}$
Rhode Island	Red	Macclesfield, NC, U.S.	2	*pti-1*$^{+}$/*pti-1*$^{+}$	*pti-2*$^{+}$/*pti-2*$^{+}$
Rhode Island	Red	Canterbury, U.K.	2	*pti-1*$^{+}$/*pti-1*$^{+}$	*pti-2*$^{+}$/*pti-2*$^{+}$
Rock	Barred	Ideal Poultry	2	*pti-1*$^{+}$/*pti-1*$^{+}$	*pti-2*$^{+}$/*pti-2*$^{+}$
Rock	White	East Lansing, MI, U.S.	1	*pti-1*$^{+}$/*pti-1*$^{+}$	*pti-2*$^{+}$/*pti-2*$^{+}$
Russian	White	Canterbury, U.K.	2	*pti-1*$^{+}$/*pti-1*$^{+}$	*pti-2*$^{+}$/*pti-2*$^{+}$
Saipan Jungle Fowl	Wild-type	East Lansing, MI, U.S.	1	*pti-1*$^{+}$/*pti-1*$^{+}$	*pti-2*$^{+}$/*pti-2*$^{+}$
Sebright	Golden	Lucasville, OH, U.S.	1	*pti-1*$^{+}$/*pti-1*$^{+}$	*pti-2*$^{+}$/*pti-2*$^{+}$
Sebright	Golden	Macclesfield, NC, U.S.	2	*pti-1*$^{+}$/*pti-1*$^{+}$	*pti-2*$^{+}$/*pti-2*$^{+}$
Sebright	Silver	Macclesfield, NC, U.S.	2	*pti-1*$^{+}$/*pti-1*$^{+}$	*pti-2*$^{+}$/*pti-2*$^{+}$
Sebright	Silver	Murray McMurray	16	*pti-1*$^{+}$/*pti-1*$^{+}$	*pti-2*$^{+}$/*pti-2*$^{+}$

Breed	Variety	Source	Number of animals	Genotype	
Shamos	Black	Ideal Poultry	1	*pti-1⁺/pti-1⁺*	*pti-2⁺/pti-2⁺*
Sicilian Buttercup	Buttercup	Ideal Poultry	2	$pti\text{-}1^{+}/pti\text{-}1^{+}$	$pti\text{-}2^{+}/pti\text{-}2^{+}$
Silkie[a]	Black	Ideal Poultry	2	*Pti-1/Pti-1*	*Pti-2/Pti-2*
Silkie[a]	Black	Raleigh, NC, U.S.	2	*Pti-1/Pti-1*	Non-fixation
Silkie[a]	Blue	East Lansing, MI, U.S.	1	*Pti-1/Pti-1*	Non-fixation
Silkie[a]	Uncharacterized	China	2	*Pti-1/Pti-1*	$pti\text{-}2^{+}/pti\text{-}2^{+}$
Silkie[a]	Uncharacterized	Ideal Poultry	2	*Pti-1/Pti-1*	Non-fixation
Silkie[a]	White	Madison, WI, U.S.	2	*Pti-1/Pti-1*	Non-fixation
Silkie[a]	White	Canterbury, U.K.	2	*Pti-1/Pti-1*	Non-fixation
Silkie[a]	White	Raleigh, NC, U.S.	2	*Pti-1/Pti-1*	*Pti-2/Pti-2*
Silkie[a]	White	Raleigh, NC, U.S.	2	*Pti-1/Pti-1*	Non-fixation
Spanish	Uncharacterized	Ames, IA, U.S.	2	$pti\text{-}1^{+}/pti\text{-}1^{+}$	$pti\text{-}2^{+}/pti\text{-}2^{+}$
Spanish	White-faced Black	Murray McMurray	1	$pti\text{-}1^{+}/pti\text{-}1^{+}$	$pti\text{-}2^{+}/pti\text{-}2^{+}$
Spitzhauben	German	Raleigh, NC, U.S.	2	$pti\text{-}1^{+}/pti\text{-}1^{+}$	$pti\text{-}2^{+}/pti\text{-}2^{+}$
Spitzhauben	Uncharacterized	Cackle Hatchery	1	$pti\text{-}1^{+}/pti\text{-}1^{+}$	$pti\text{-}2^{+}/pti\text{-}2^{+}$
Sultan[a]	Uncharacterized	Canterbury, U.K.	2	Non-fixation	*Pti-2/Pti-2*
Sultan[a]	White	East Lansing, MI, U.S.	1	*Pti-1/Pti-1*	Non-fixation
Sultan[a]	White	Macclesfield, NC, U.S.	2	*Pti-1/Pti-1*	*Pti-2/Pti-2*
Sultan[a]	White	Raleigh, NC, U.S.	2	*Pti-1/Pti-1*	*Pti-2/Pti-2*
Sumatra	Black	Lucasville, OH, U.S.	2	$pti\text{-}1^{+}/pti\text{-}1^{+}$	$pti\text{-}2^{+}/pti\text{-}2^{+}$
Sumatra	Black	Raleigh, NC, U.S.	2	$pti\text{-}1^{+}/pti\text{-}1^{+}$	$pti\text{-}2^{+}/pti\text{-}2^{+}$
Sumatra	Blue	Lucasville, OH, U.S.	2	$pti\text{-}1^{+}/pti\text{-}1^{+}$	$pti\text{-}2^{+}/pti\text{-}2^{+}$
Sumatra	Blue	Marshfield, WI, U.S.	1	$pti\text{-}1^{+}/pti\text{-}1^{+}$	$pti\text{-}2^{+}/pti\text{-}2^{+}$
Sumatra	Uncharacterized	Murray McMurray	1	$pti\text{-}1^{+}/pti\text{-}1^{+}$	$pti\text{-}2^{+}/pti\text{-}2^{+}$
Sumatra	Uncharacterized	Lucasville, OH, U.S.	1	$pti\text{-}1^{+}/pti\text{-}1^{+}$	$pti\text{-}2^{+}/pti\text{-}2^{+}$
Sussex	Light	Canterbury, U.K.	2	$pti\text{-}1^{+}/pti\text{-}1^{+}$	$pti\text{-}2^{+}/pti\text{-}2^{+}$
Sussex	Speckled	Macclesfield, NC, U.S.	2	$pti\text{-}1^{+}/pti\text{-}1^{+}$	$pti\text{-}2^{+}/pti\text{-}2^{+}$
Svarthöna	Black	Uppsala, Sweden	2	$pti\text{-}1^{+}/pti\text{-}1^{+}$	$pti\text{-}2^{+}/pti\text{-}2^{+}$
Ukrainian	Muffed	Canterbury, U.K.	2	$pti\text{-}1^{+}/pti\text{-}1^{+}$	$pti\text{-}2^{+}/pti\text{-}2^{+}$
Uzbek Game	Uncharacterized	Canterbury, U.K.	2	$pti\text{-}1^{+}/pti\text{-}1^{+}$	$pti\text{-}2^{+}/pti\text{-}2^{+}$
Welsummer	Uncharacterized	Lucasville, OH, U.S.	1	$pti\text{-}1^{+}/pti\text{-}1^{+}$	$pti\text{-}2^{+}/pti\text{-}2^{+}$
Wyandotte	Chocolate	GreenFire Farms	2	$pti\text{-}1^{+}/pti\text{-}1^{+}$	$pti\text{-}2^{+}/pti\text{-}2^{+}$
Wyandotte	Partridge	Murray McMurray	2	$pti\text{-}1^{+}/pti\text{-}1^{+}$	$pti\text{-}2^{+}/pti\text{-}2^{+}$
Wyandotte	Silver penciled	Murray McMurray	2	$pti\text{-}1^{+}/pti\text{-}1^{+}$	$pti\text{-}2^{+}/pti\text{-}2^{+}$
Yokohama	Red Shoulder	Ideal Poultry	1	$pti\text{-}1^{+}/pti\text{-}1^{+}$	$pti\text{-}2^{+}/pti\text{-}2^{+}$
Yokohama	Red shoulder	Lucasville, OH, U.S.	1	$pti\text{-}1^{+}/pti\text{-}1^{+}$	$pti\text{-}2^{+}/pti\text{-}2^{+}$
Аврóра	Blue	Canterbury, U.K.	2	$pti\text{-}1^{+}/pti\text{-}1^{+}$	$pti\text{-}2^{+}/pti\text{-}2^{+}$
		Total	295		

[a] Chicken samples with feathered leg phenotype. Others express clean leg
