# Supplementary material for: Mutations Upstream of the TBX5 and PITX1 Transcription Factor Genes Are Associated with Feathered Legs in the Domestic Chicken
Source: Mol Biol Evol. 2020 Apr 28;37(9):2477–86. doi: 10.1093/molbev/msaa093 (PMC7475036; doi:10.1093/molbev/msaa093)
Supplement: msaa093_Supplementary_Data [file FigureS1.pdf › msaa093-Suppl_Data/FigureS1.pdf]

a

```
Human.chr12:   114454550 aaactttcatgtctgatgaactttcatgtctgggaagaccaacaaaggct 114454599
               <<<<<<<<< |||| | ||| ||   | |    || |  || ||||||  ||  ||   | <<<<<<<<<
Chicken.chr15: 012573200 aaacatccatctccagtaatgagtcct--ctaggaaga--aatgaaaaat 012573155

               114454600 accaagggggcaaagtctggagaagtcaattaggtgggcttgcaataaga 114454649
               <<<<<<<<< | |       | | | ||||  |||||||||| || |  ||  ||||||| <<<<<<<<<
               012573154 agc-------ccaggcctggtaaagtcaattaagttgcttttaaataaga 012573112

               114454650 ggttattctgagaggccaggagttaacatagaaagtcttgtcaccaaag- 114454698
               <<<<<<<<< |||||  | ||||||||| ||||||||||  |||| ||| |  |||  |  <<<<<<<<<
               012573111 ggttacccagagaggccaagagttaacatgtaaaggcttattgccagtgc 012573062

               114454699 ----ggaactagaagaaggatcttttagaccgggggggacatttggttgc 114454744
               <<<<<<<<<       || || ||| |||     ||  |     |  ||||||||||||| <<<<<<<<<
               012573061 tgtaaaaaataaaaggagg----cttgaatatttgaagacatttggttgc 012573016

               114454745 tattatgaatgaacaaacccagcaaagctgaaaca-----ggaaaaacgg 114454789
               <<<<<<<<< |||||| ||  |    | | |  |  |  |  |||      ||  ||| | <<<<<<<<<
               012573015 tattataaacaagtggatctaatagtgtgggtacattcatagactaactg 012572966

               114454790 ca-atgttgagcatctcagtgggcta 114454814
               <<<<<<<<<  | | |||| |||||| |   ||  | <<<<<<<<<
               012572965 tagaagttgggcatcttataaggaca 012572940
```

b

```
Human.chr5:    135435115 cctttcattcttggggtctcattctacaacccgatggccaaggcagacag 135435164
               <<<<<<<<< || | | ||||| || ||   |||| ||  | ||   | ||  |||  |  <<<<<<<<<
Chicken.chr13: 016099329 ccctgccttctttggttcatgttct-cattctgaccacaaatacagcaaa 016099281

               135435165 caccgtccgctggccagggggtggggttaattcctgcaaggacctgggtg 135435214
               <<<<<<<<<   | |  |     |  ||  | || ||||||  ||    |  ||       <<<<<<<<<
               016099280 agctgatctgaaacttggatg-ggtgttaataactaatggaccccaacaa 016099232

               135435215 aaggtctgcctggccaaggcag-cccgttgcaaggtttgaatcatggctg 135435263
               <<<<<<<<< |  |||||     | ||||  |  | | || | |||| | || |  |    <<<<<<<<<
               016099231 atcgtctgaaatacaaaggtgggtcagatgaatggttcgtataaaagaaa 016099182

               135435264 tctcagctgggccatcgggtgggaattaaaatttaatttagaaagtcctg 135435313
               <<<<<<<<<    | | ||  ||||| |||||||||||||||||||||||||| || ||  <<<<<<<<<
               016099181 ctcccggtgaaccatcaggtgggaattaaaatttaatttagaatgtacta 016099132

               135435314 tctctgcggccctggtggccgtcgt---ctgggtgattgactggctggtt 135435360
               <<<<<<<<< || |||| ||  ||||||  || ||    |    ||||||||| |||||| <<<<<<<<<
               016099131 tcactgctgcggtggtggtggtggtgatttacttgattgactg-ctggtt 016099083

               135435361 tctttcacctttgcacgtcttgggtttttactggcagaggaaatgaaggc 135435410
               <<<<<<<<< ||||||||||||||| | ||| ||||||||||  | |||||||| ||||| <<<<<<<<<
               016099082 tctttcacctttgcaagccttaggtttttactatctgaggaaattaaggc 016099033

               135435411 tgaactggggcaggaaattccatgtacacaggtgtttctgattagccccg 135435460
               <<<<<<<<< |||| ||  |||||||||||| ||||| |||||||||||| ||| ||    <<<<<<<<<
               016099032 tgaattgcagcaggaaattccttgtacccaggtgtttctggttatccgat 016098983

               135435461 t-gcacctcacc--caggggcacaacca-ggggctgagatg--------c 135435498
               <<<<<<<<< |   | |||| |   ||||   |||  |   || || ||||          <<<<<<<<<
               016098982 taataactcagcagtagggaggcaaggattaggatgggatggtttggaat 016098933

               135435499 ctgcatga-gcccatgggcccctctctggcttggcaactgtggcagc--- 135435544
               <<<<<<<<< ||  ||||  | ||| |  |  | ||   | | |  | | |||||||    <<<<<<<<<
               016098932 ctaaatgattctcattgctcagtatcacacattgacattctggcagctgc 016098883

               135435545 ----tttgcatctagggaaggctgg 135435565
               <<<<<<<<<     ||||     || ||| | ||| <<<<<<<<<
               016098882 tttgtttgttctcagtgaaagatgg 016098858
```
